# Supplementary material for: Di-berberine conjugates as chemical probes of Pseudomonas aeruginosa MexXY-OprM efflux function and inhibition
Source: NPJ Antimicrob Resist. 2023 Oct 6;1:12. doi: 10.1038/s44259-023-00013-4 (PMC11721654; doi:10.1038/s44259-023-00013-4)
Supplement: Supplementary file 1 — Supplementary Materials [file 44259_2023_13_MOESM1_ESM.pdf]

Supplementary Materials For:

Di-berberine conjugates as chemical probes of *Pseudomonas aeruginosa* MexXY-OprM efflux function and inhibition

Logan G. Kavanaugh[1#], Andrew R. Mahoney[2#], Debayan Dey[1], William M. Wuest[2,3*] and Graeme L. Conn[1,3*]

[#]Co-first authors

*Correspondence to: Graeme L. Conn, gconn@emory.edu; or, William M. Wuest, wwuest@emory.edu

This file contains:

- Supplementary Tables 1-6
- Supplementary Figures 1-10
- Supplementary Methods: Details of chemical synthesis and compound analysis
- Supplementary References
- Appendix: ^{1}H and ^{13}C NMR spectra for all synthesized intermediates and berberine analogs.

Supplementary Tables

**Supplementary Table 1. Strains used in this study.**		
Strain	Characteristics	Source (Reference)
PAO1 (K767)	Wild-type PAO1;<br>lab evolved strain	Poole Lab<br>(Sobel et al. 2003)[1]
PAO1Δ*mexXY*<br>(K1525)	K767Δ*mexXY*	Poole Lab<br>(Sobel et al. 2003)[1]
K2156	Pan-aminoglycoside resistant<br>clinical isolate	Poole Lab<br>(Sobel et al. 2003)[1]
K2161	Pan-aminoglycoside resistant<br>clinical isolate	Poole Lab<br>(Sobel et al. 2003)[1]
PA14	Virulent clinical isolate	Goldberg Lab
PA7	MDR clinical isolate	Goldberg Lab

Supplementary Table 2. SP Glide docking scores for berberine and analogs in the *P. aeruginosa* PAO1, PA7, and PA14 MexY binding pocket.

Compound	SP Glide Docking score (Average)[1,2]			
	$MexY^{PAO1}$		$MexY^{PA7}$	$MexY^{PA14}$
	6IOL (Chain F)	6TA6 (Chain L)	6IOL (Chain F)	6IOL (Chain F)
Berberine	-6.46 (-6.04)	-5.70 (-5.38)	-6.41 (-6.13)	-5.75 (-5.01)
Ber-Pip [**1**]	-6.03 (-5.82)	-5.82 (-5.20)	-5.74 (-4.41)	-5.02 (-4.94)
Ber-Carb [**2**]	-4.73 (-4.68)	-5.75 (-5.75)	-5.41 (-3.37)	-4.23 (-4.00)
Ber-Prop [**3**]	-8.10 (-7.97)	-8.36 (-8.01)	-8.17 (-7.99)	-7.87 (-7.44)
Ber-C6 [**4**]	-8.99 (-7.39)	-9.32 (-8.79)	-9.33 (-7.74)	-8.10 (-6.72)
Ber-C3 [**5**]	-9.18 (-7.74)	-9.28 (-8.97)	-9.77 (-7.47)	-8.37 (-5.85)
Ber-C4 [**6**]	-8.88 (-7.51)	-9.50 (-9.11)	-9.06 (-6.68)	-8.09 (-6.21)
Ber-C8 [**7**]	-9.28 (-7.38)	-9.32 (-8.74)	-10.30 (-8.37)	-8.48 (-6.91)
Ber-C10 [**8**]	-9.55 (-7.35)	-9.77 (-9.13)	-9.73 (-8.23)	-8.63 (-7.22)
Ber-C12 [**9**]	-9.77 (-7.14)	-9.53 (-8.71)	-10.10 (-7.80)	-8.45 (-6.99)
Ber-pAr [**10**]	-9.37 (-7.67)	-9.56 (-9.29)	-10.01 (-6.47)	-8.60 (-6.96)
Ber-Biph [**11**]	-10.36 (-10.39)	-10.26 (-9.65)	-11.08 (-10.23)	-10.56 (-10.26)
Ber-PEG5 [**12**]	-9.73 (-7.76)	-9.43 (-9.13)	-9.87 (-7.52)	-8.70 (-6.80)
Ber-PEG8 [**13**]	-9.90 (-7.89)	-9.75 (-9.36)	-10.10 (-7.72)	-8.94 (-7.47)

[1]Docking scores correspond to the top scored pose (shown in figures, where applicable) and the average of all docking poses for each compound in parenthesis.
[2]The PDB code noted (with chain ID in parenthesis) indicates to the structure used to generate the $MexY^{PAO1}$, $MexY^{PA7}$, and $MexY^{PA14}$ homology models used for docking studies (see Methods for details).

Supplementary Table 3. Contact residues for berberine and analogs docked in the PBP/ DBP of MexY from *P. aeruginosa* strains PAO1, PA7, and PA14 using SP Glide.

Ligand	PAO1	PA7	PA14
Berberine	R166, W177, F610	Y127, F610	Q175, K291
Ber-Pip [**1**]	Y127	Y127, K173	S88, K291
Ber-Carb [**2**]	K173, K291	K173, K291	K173
Ber-Prop [**3**]	D124	D124	D133,K291,Y326
Ber-C6 [**4**]	T176, E273, Y752, K764	T176, K273, Y752, K764	S46, Y127, S272, E273, K291, K764
Ber-C3 [**5**]	S46, Y127, T176, S272, E273, K291	Y127, E175, K291	N44, D133, K291
Ber-C4 [**6**]	E175	Y127, F276	N44, Y127, K291
Ber-C8 [**7**]	S46, K79	Y127, K173, T176, K291	R116, S272, E273, K291
Ber-C10 [**8**]	E175, K291	E175, E273, K764	N44, S272, E273, K764
Ber-C12 [**9**]	Y127, K291	E175, K291, K764	E273, K291
Ber-pAr [**10**]	Y127, K173, S671	K173, E175, K291	S46
Ber-Biph [**11**]	K173, E175, E273, K291	K173, E175, E273, K291	K173, K291
Ber-PEG5 [**12**]	Y127, S671	Y127, S671	K173, Q175, K291
Ber-PEG8 [**13**]	Y127, E175	S88, Y127,F276, K291, S671	S46, S88, Y127, D133, Q175, E273

Supplementary Table 4. Predicted minimum inhibitory concentration (MIC) and fractional inhibitory concentration (FIC) for berberine and analogs.

	Compound	Predicted MIC (μg/mL)[1]	Antibiotic[2]	FIC	
				Range	Average
Generation 1	Berberine	979	Kan	0.50	0.50
			Gen	0.50	0.50
	Ber-Pip [**1**]	3549	Kan	0.50	0.50
			Gen	1.14	1.14
	Ber-Carb [**2**]	760	Kan	1.67	1.67
			Gen	0.52	0.52
	Ber-Prop [**3**]	947	Kan	0.39-0.50	0.44
			Gen	0.39-0.50	0.44
	Ber-C6 [**4**]	287	Kan	0.28-0.51	0.38
			Gen	0.36-0.51	0.48
Generation 2	Ber-C3 [**5**]	584	Kan	0.30-0.50	0.40
			Gen	0.30-0.50	0.38
	Ber-C4 [**6**]	508	Kan	0.38-0.5	0.44
			Gen	0.38-0.5	0.44
	Ber-C8 [**7**]	250	Kan	0.28-0.5	0.39
			Gen	0.38-0.5	0.40
	Ber-C10 [**8**]	200	Kan	0.17-0.51	0.31
			Gen	0.17-0.51	0.33
	Ber-C12 [**9**]	180	Kan	0.26-0.51	0.36
			Gen	0.26-0.77	0.46
Generation 3	Ber-pAr [**10**]	400	Kan	0.52-0.57	0.55
			Gen	0.33-0.52	0.43
	Ber-Biph [**11**]	502	Kan	0.50-0.51	0.51
			Gen	0.52	0.52
	Ber-PEG5 [**12**]	545	Kan	0.56	0.56
			Gen	0.48-0.53	0.51
	Ber-PEG8 [**13**]	622	Kan	0.55	0.55
			Gen	0.46-0.53	0.49

[1]Predicted berberine analog MIC was determined by plotting final OD_{600} values from cultures at 18 hours after exposure to a range of ligand concentrations (128-0 μg/mL) and using nonlinear regression of the log transformed OD_{600} values to obtain the MIC value (y intercept = 0).
[2]Aminoglycosides: kanamycin (Kan) and gentamicin (Gen).

Supplementary Table 5. Three-way synergy assay with berberine, Ber-C3 [5], and amikacin.

Berberine (µg/mL)	Ber-C3 [**5**] (µg/mL)	Ami MIC (µg/mL)
0	0	1
	64	0.25
64	0	0.5
	1	1
	2	1
	4	1
	8	1
	16	1
	32	0.5
	64	0.5
	128	0.25

Supplementary Table 6. Contact residues for berberine and select analogs in the PBP and DBP of *P. aeruginosa* PAO1 MexY before and after MD simulation.

Ligand	Before MD (0 ns)[1]	After MD (50 ns)[2]
Berberine	-	F610
Ber-C3 [**5**]	R116, Y127, K173, E175, K291, F610	R67, D124, **E175**, F276, **K291**, **F610**, S671, K764
Ber-C12 [**9**]	Y127, Q163, R763	K291, Y326
Ber-pAr [**10**]	R166, E273, F276, F610, S671, Q761	R67, **F276**, K291, **F610**, **S671**

[1]The predicted interactions noted are after the initial equilibration (10 ns) period immediately prior to the 50 ns MD production run, and may therefore differ from initial docking (**Supplemental Table 1**).

[2]Interactions with residues in bold are present before and after the MD simulation.

Supplementary Figures

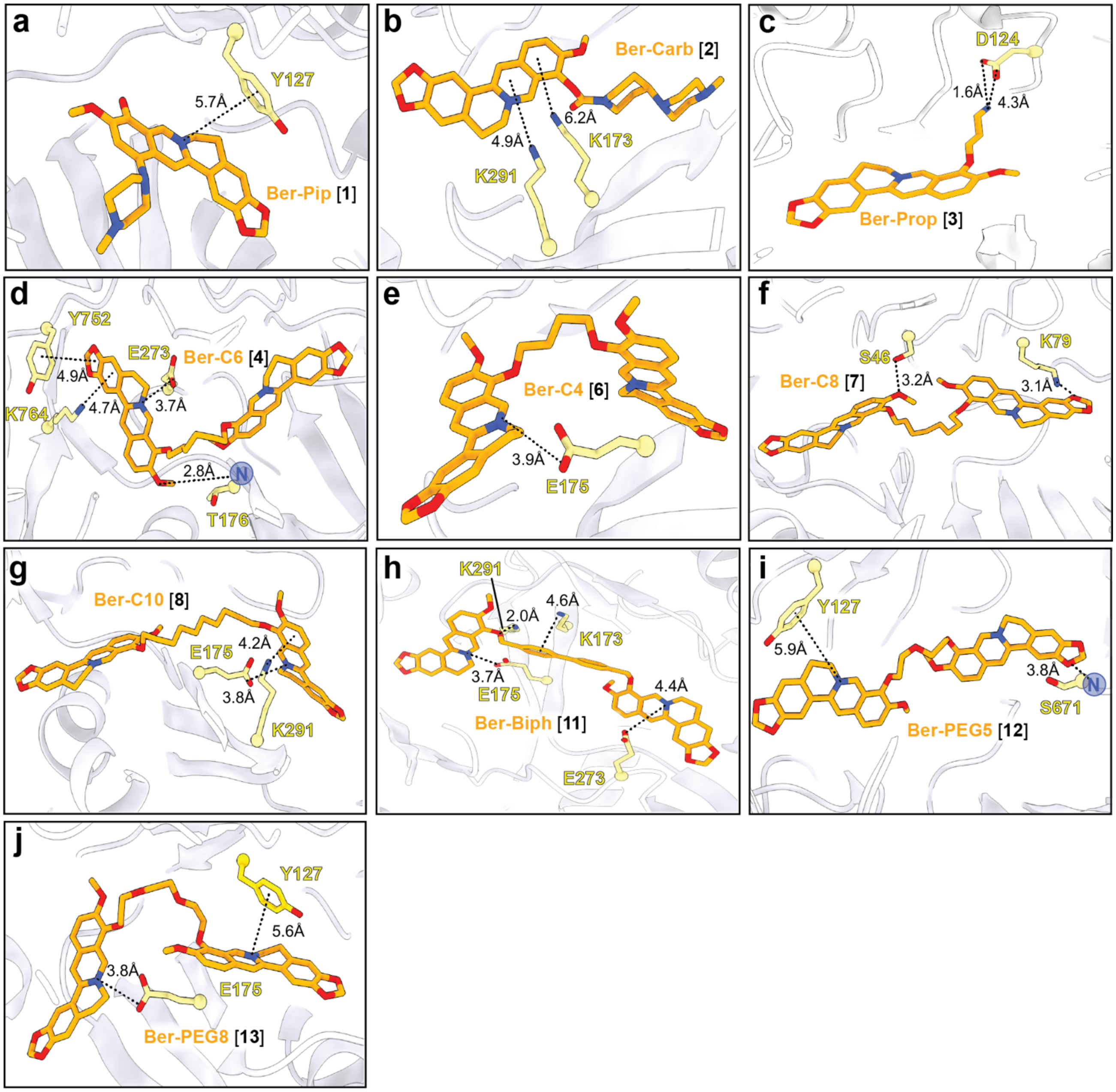

Supplementary Figure 1. Lowest energy docking poses of berberine analogs in the MexY^{PAO1} binding pocket. Docking of all berberine analogs (orange) not included in **Fig.2**. *Generation 1* analogs: **(a)** Ber-Pip [**1**], **(b)** Ber-Carb [**2**], **(c)** Ber-Prop [**3**], **(d)** Ber-C6 [**4**]. All *Generation 1* analogs docked preferentially in the MexY^{PAO1} DBP, with Ber-C6 [**4**] docked in the DBP at the PC1/DN interface. *Generation 2* analogs: **(e)** Ber-C4 [**6**], **(f)** Ber-C8 [**7**], and **(g)** Ber-C10 [**8**]. Ber-C4 [**6**] and Ber-C10 [**8**] showed preference for the DBP. In contrast, Ber-C8 [**7**] showed preference for the PBP. *Generation 3* analogs:**(h)** Ber-Biph [**11**], **(i)** Ber-PEG5 [**12**], and **(j)** Ber-PEG8 [**13**]. Ber-Biph [**11**] and Ber-PEG8 [**13**] showed preference for the DBP, while Ber-PEG5 [**12**] spanned the PBP to DBP.

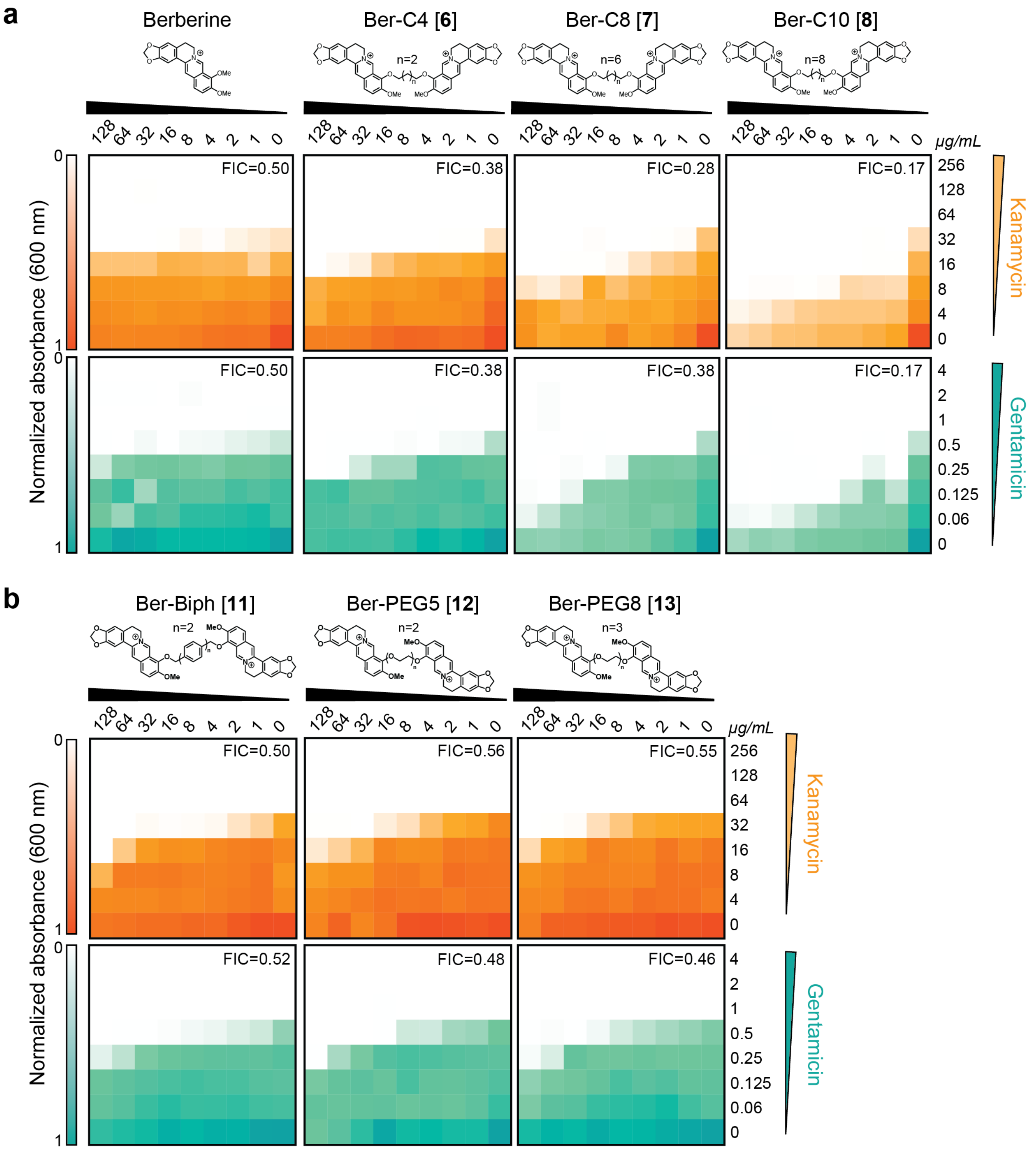

Supplementary Figure 2. Checkerboard synergy assays for additional di-berberine conjugates. Checkerboard synergy assays in *P. aeruginosa* PAO1 with selected analogs (those not shown in **Fig. 1** and **Fig. 3**): **(a)** berberine (same as shown in other figures but included here for ease of comparison) and *Generation 2* ligands Ber-C4 [**6**], Ber-C8 [**7**], and Ber-C10 [**8**]. Increased synergy compared to berberine is observed for all tested compounds with both Kan (top, orange) and Gen (bottom, teal). **(b)** *Generation 3* compounds Ber-Biph [**11**], Ber-PEG5 [**12**], and Ber-PEG8 [**13**], which all showed similar synergy with Kan (top, orange) and Gen (bottom, teal) as berberine. All data are shown as the normalized mean of the optical density (OD_{600}) of two biological replicates, (0 is no growth, 1 is maximum growth). The lowest FIC score for each compound-antibiotic pairing is given in the upper, right corner.

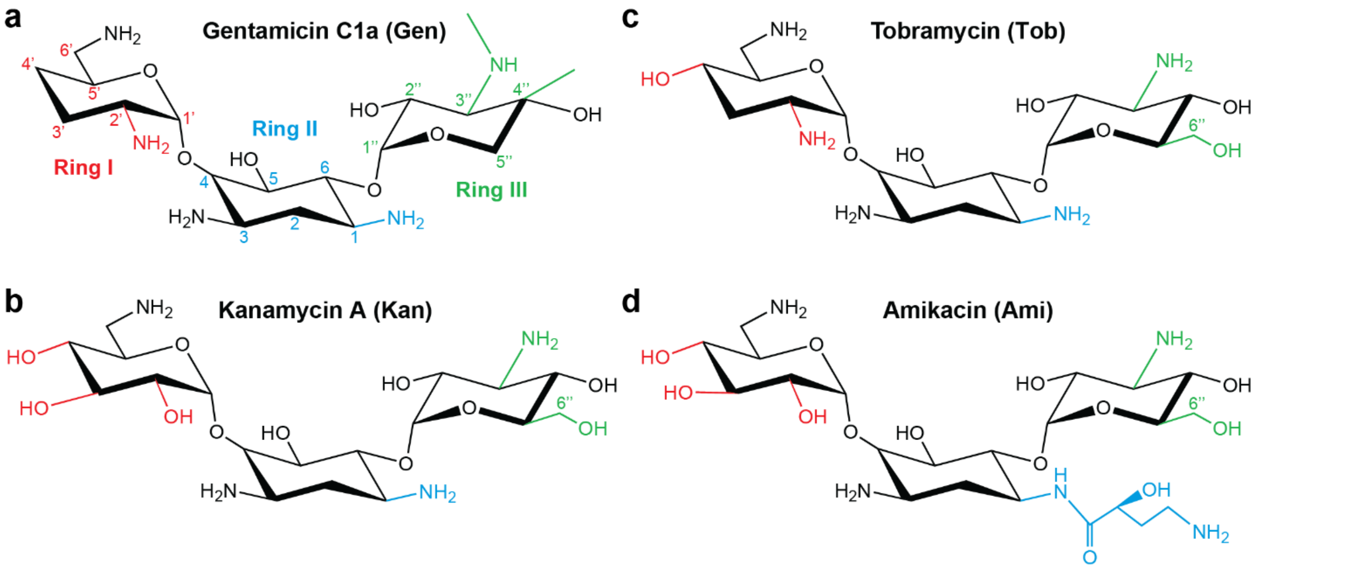

Supplementary Figure 3. Structures of aminoglycosides used in this study. Chemical structures of (**a**) gentamicin C1a (Gen), (**b**) kanamycin A (Kan), (**c**) tobramycin (Tob), and (**d**) amikacin (Ami), highlighting the differences between the four structures color coded by ring. Ring and carbon atom numbers within each aminoglycoside are indicated on the structure of gentamicin (except Ring III 6'' which is only present in the three other aminoglycosides). The deoxystreptamine ring (Ring II) is appended with a (S)-4-amino-2-hydroxubutyrate group on the amino group of position 1 (commonly referred to as L-HABA, or, L-hydroxyaminobuteroyl amide).

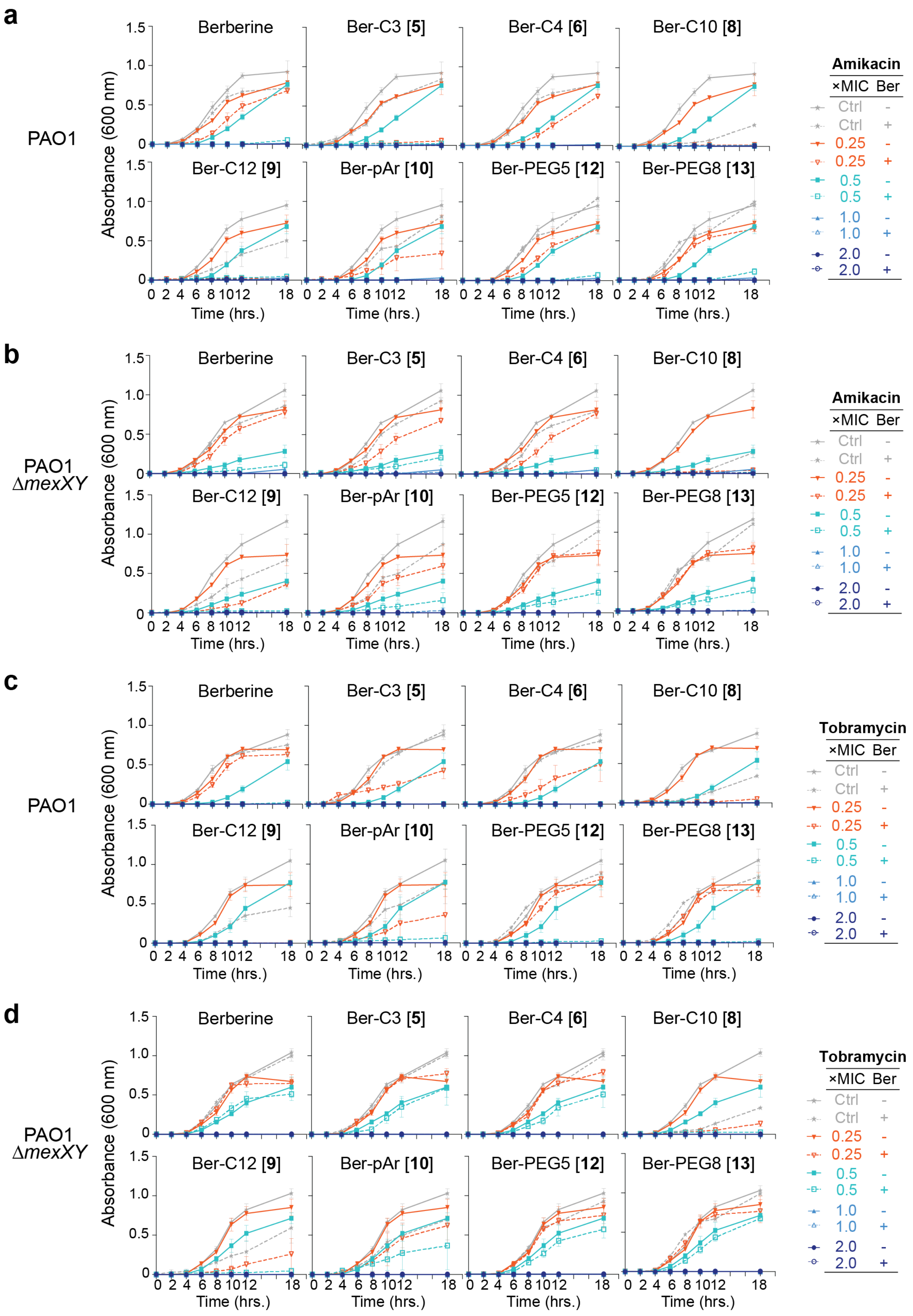

Supplementary Figure 4. (See next page for full legend.)

Supplementary Figure 4. Growth curves with berberine and select analogs with amikacin and tobramycin in *P. aeruginosa* PAO1 and isogenic PAO1Δ*mexXY*. Time-kill assays for berberine and analogs from *Generation 2* (Ber-C3 [**5**], Ber-C4 [**6**], Ber-C10 [**8**], and Ber-C12 [**9**]) and *Generation 3* (Ber-pAr [**10**], Ber-PEG5 [**12**], and Ber-PEG8 [**13**]) at 64 µg/mL are shown with (**a,b)** Ami and **(c,d)** Tob over 18 hours in both *P. aeruginosa* PAO1 and its corresponding *mexXY* isogenic knockout (PAOΔ*mexXY*). Data are shown as the average OD_{600} of biological replicates, each with two technical replicates; error bars are standard deviation.

a

Compound	RBC Hemolysis
Berberine	>128 µg/mL
Ber-C4 [**6**]	>128 µg/mL
Ber-C10 [**8**]	>128 µg/mL
Ber-C12 [**9**]	>128 µg/mL
Ber-pAr [**10**]	>128 µg/mL
Triton-X (+)	<1%

b

Conc. (µg/mL)	PAO1		PAO1Δ*mexXY*	
Ber-C12 [**9**]	0	64	0	64
Vancomycin	128	128	128	128

c

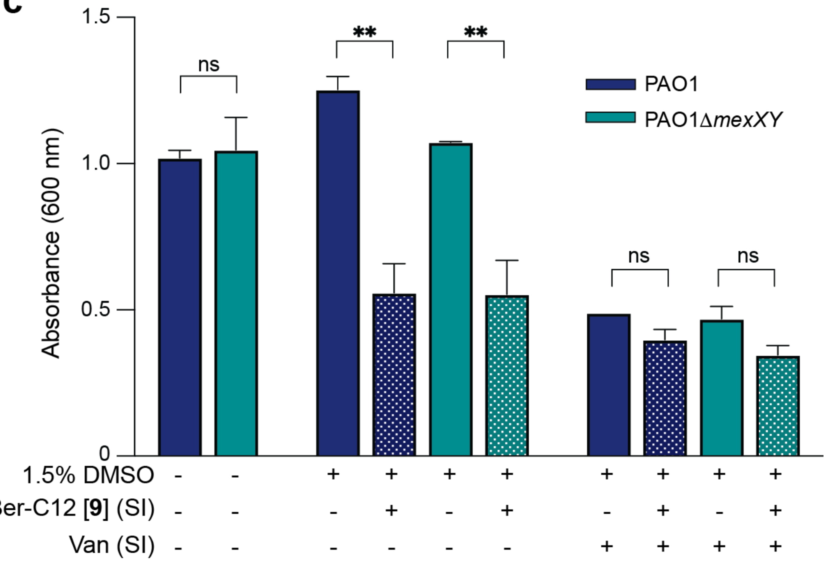

Supplementary Figure 5. Berberine and di-berberine conjugates do not disrupt *P. aeruginosa* membrane. **(a)** Hemolysis assay was performed with sheep red blood cells in the presence of 128-0 µg/mL of berberine and berberine analogs (Ber-C4 [**6**], Ber-C10 [**8**], Ber-C12 [**9**], and Ber-pAr [**10**]). The reported concentration of berberine analog or Triton-X (positive control) is that needed to lyse ≥ 20% of red blood cell as determined by absorbance measurement (OD_{540}). Berberine and analogs did not lyse ≥ 20% of red blood cells at any concentration tested (up to 128 µg/mL). The average from triplicate assays are reported. **(b)** The vancomycin (Van) MIC was determined in *P. aeruginosa* PAO1 and *P. aeruginosa* PAO1Δ*mexXY* in the absence and presence of 64 µg/mL of Ber-C12 [**9**] and did not change under any conditions tested. The assay was performed in duplicate. **(c)** Final absorbance (OD_{600}) for PAO1 (blue) and PAO1Δ*mexXY* (teal) in the absence (filled bar) and presence (stippled bar) of 64 µg/mL Ber-C12 [**9**] and a sub-inhibitory concentration (SI) of Van (64 µg/mL). Data are plotted as average and error bars represent the standard deviation. Statistical significance was determined by unpaired T-test between each condition (* $p \leq 0.05$, ** $p \leq 0.01$, *** $p \leq 0.001$, and **** $p \leq 0.0001$). A difference was observed in the final absorbance for both PAO1 and PAO1Δ*mexXY* in the presence of 64 µg/mL, but this difference was not apparent upon the addition of SI of Van.

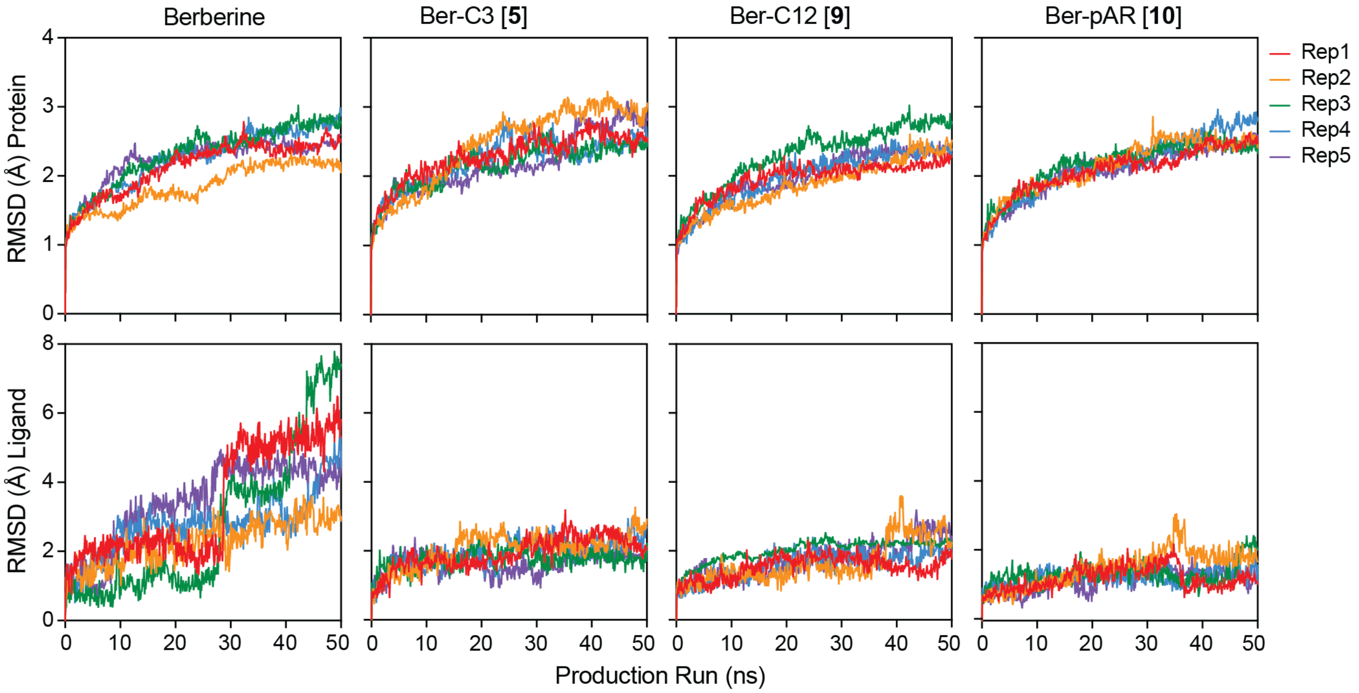

Supplementary Figure 6. Protein and ligand RMSD plots from replicate MD similations of MexY with berberine and select analogs. RMSD during the five replicate 50 ns MD simulations (Rep1-5) for the protein (top)-ligand (bottom) pairs indicated above each pair of plots: berberin, Ber-C3 [**5**], Ber-C12 [**9**], and Ber-pAR [**10**].

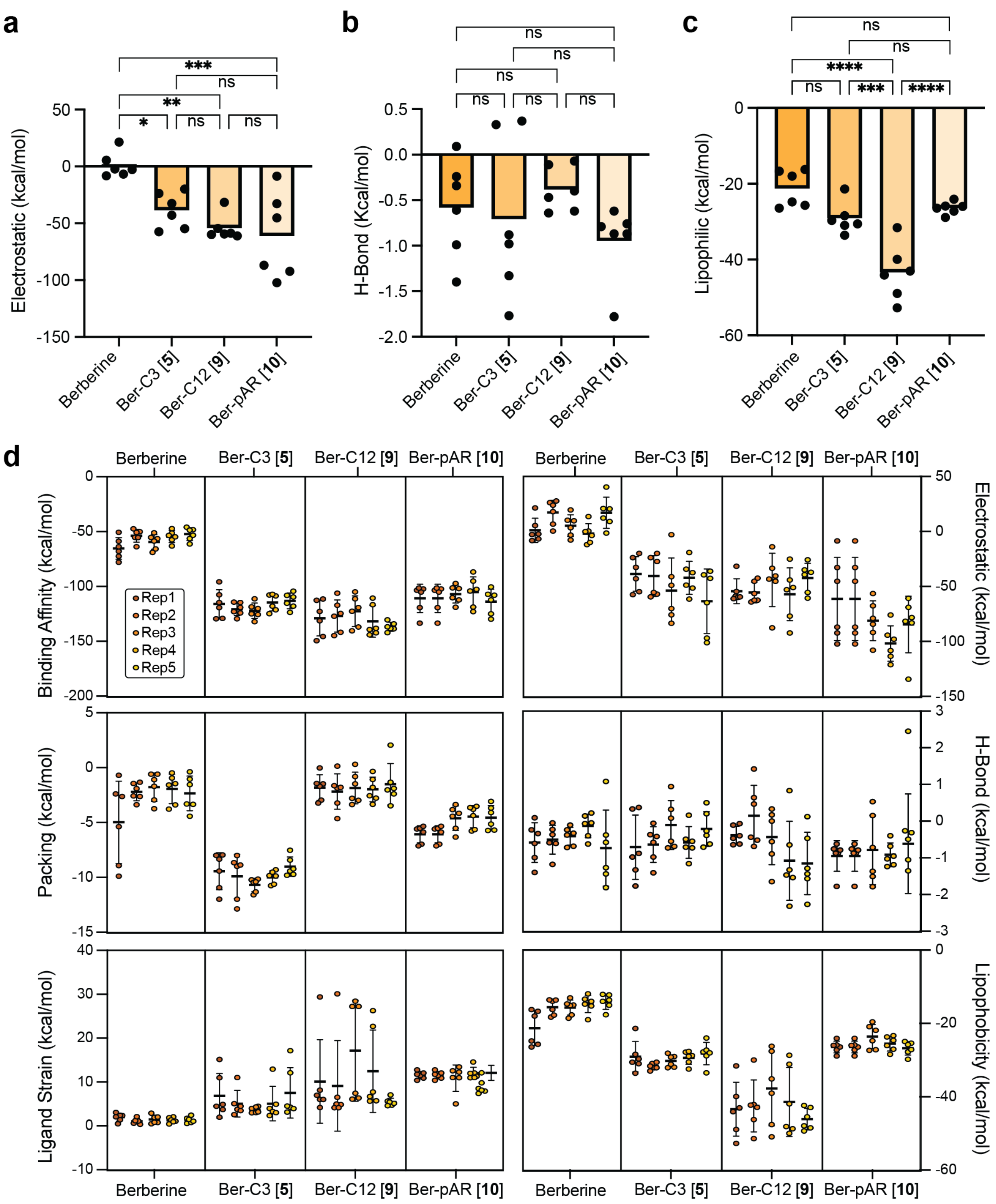

Supplementary Figure 7. MM/GBSA calculations from replicate MD simulations. Additional parameters derived from MM/GBSA calculations for MD Rep1 (also see **Fig.4**): **(a)** electrostatic interaction, **(b)** hydrogen bonding (H-bond), and **(c)** lipophilic interaction. Data shown are the individual calculated values for six frames (one per 10 ns from 0-50 ns) with the average shown as a bar plot. Statistical analysis was performed using one-way ANOVA on the mean for each ligand and a post-hoc Turkey test was used for pairwise comparison. Statistically significant results are shown (* $p \leq 0.05$, ** $p \leq 0.01$, *** $p \leq 0.001$, and **** $p \leq 0.0001$). **(d)** Summary of all MM/GBSA calculations for the five replicate simulations (Rep1-5) with berberine and top di-berberine conjugates. Data shown are the individual calculated values for six frames as for panels (a)-(c).

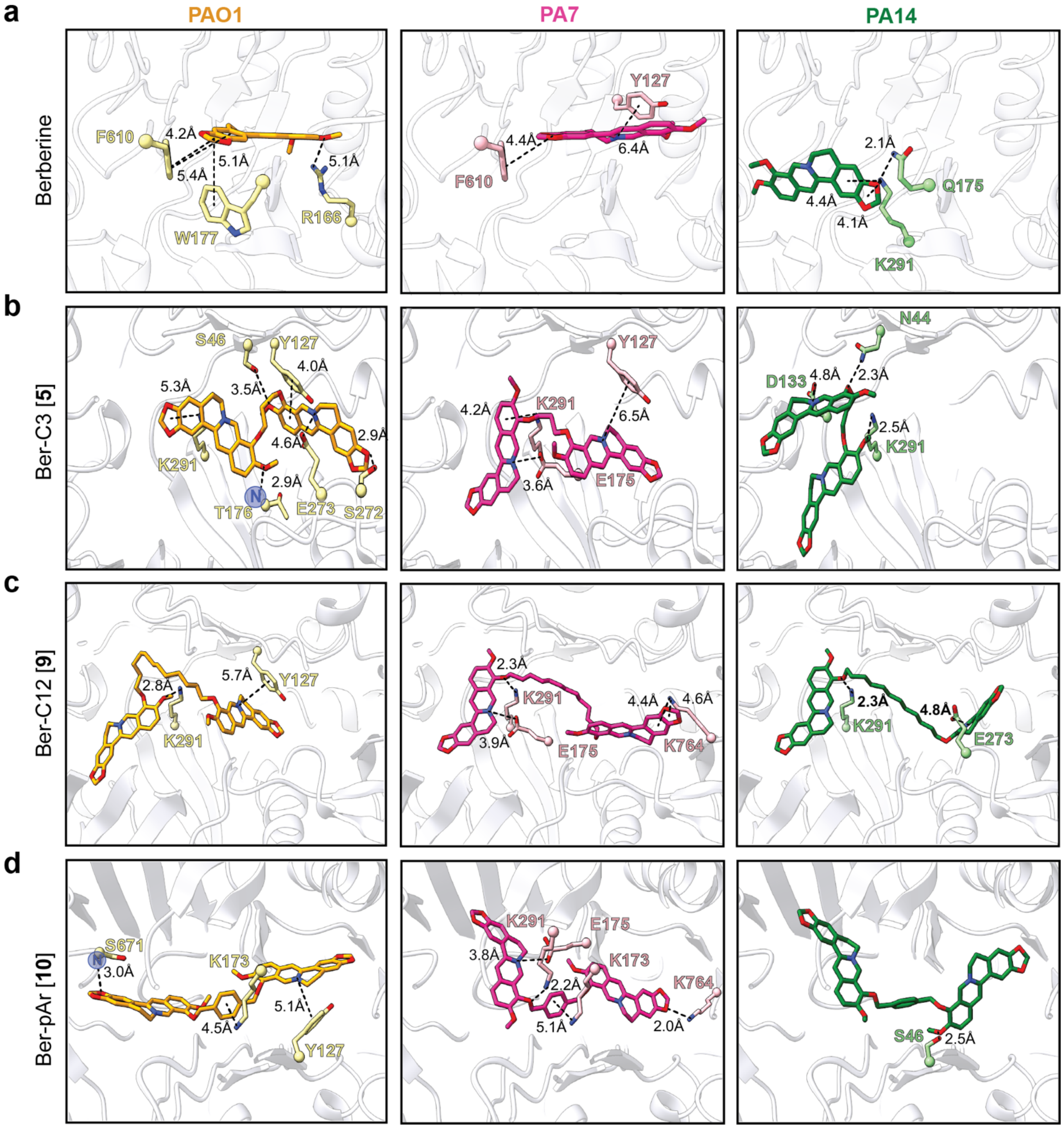

Supplementary Figure 8. Preferred docking conformation for berberine and select analogs in the binding pockets of MexY^{PAO1}, MexY^{PA7}, and MexY^{PA14}. Docking of **(a)** berberine, and di-berberine conjugate analogs **(b)** Ber-C3 [**5**], **(c)** Ber-C12 [**9**], and **(d)** Ber-pAr [**10**] in the PBP/DBP of MexY^{PAO1} (orange, left), MexY^{PA7} (pink, middle), and MexY^{PA14} (green, right). Berberine and Ber-C3 [**5**] adopt similar conformations in MexY^{PAO1}/ MexY^{PA7}. In contrast, Ber-C12 [**9**] and Ber-pAr [**10**] adopt similar conformations in MexY^{PA7}/ MexY^{PA14}.

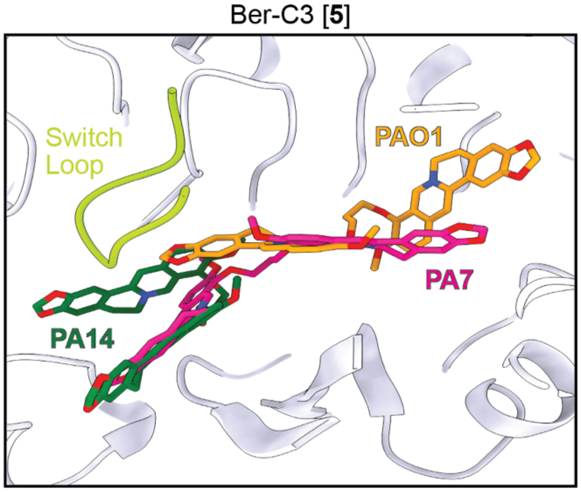

Supplementary Figure 9. Distinct predicted docking conformations of Ber-C3 [5] in the binding pocket of MexY from different *P. aeruginosa* strains. Predicted docking shows that Ber-C3 [**5**] preferentially binds in the DBP of MexY in an 'extended' conformation in MexY^{PAO1} (orange) and MexY^{PA7} (pink), but in a 'compact' conformation in MexY^{PA14} (green).

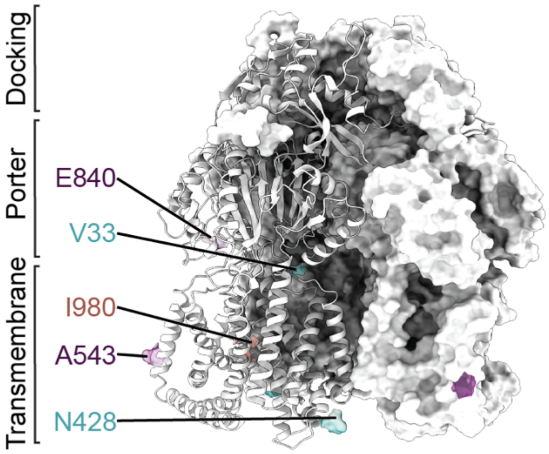

Supplementary Figure 10. Unique amino acid changes in pan-aminoglycoside resistant *P. aeruginosa* clinical isolate K2161. Unique residues identified from alignment of MexY sequences from *P. aeruginosa* PAO1 and clinical isolates K2156 (salmon) and K2161 (teal) mapped on a cartoon of MexY^{PAO1} in the binding state. Residues with the same amino acid substitution in both K2156 and K2161 are shown in purple. The three domains of the MexY transporter (docking, porter, and transmembrane) are indicated.

Supplementary Methods: Details of chemical synthesis and compound analysis

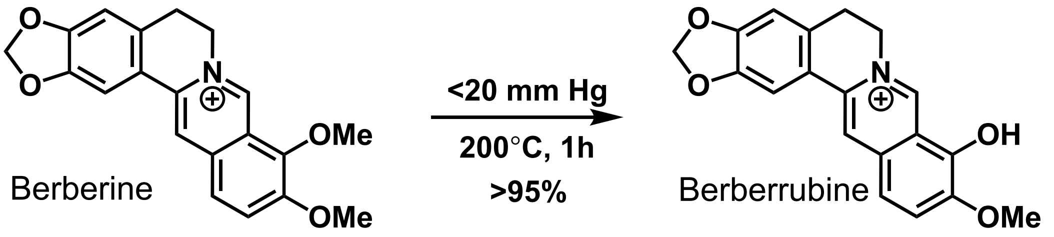

Berberrubine chloride.[2] Berberine chloride (500 mg, 1.34 mmol, 1 eq.) was heated to 200°C under vacuum (<20 mm Hg) for 45 minutes with stirring, causing a color change from yellow to red. Solid was filtered, washing with chloroform. Filtrate was purified by silica column chromatography (0-10% MeOH in DCM) to yield the title compound as a dark red solid (480 mg, >95% yield). **^{1}H NMR** (600 MHz, Chloroform-*d*) δ 9.16 (s, 1H), 7.54 (s, 1H), 7.21 (d, *J* = 7.9 Hz, 1H), 7.20 (s, 1H), 6.71 (s, 1H), 6.46 (d, *J* = 7.9 Hz, 1H), 6.02 (s, 2H), 4.36 (s, 2H), 3.87 (s, 3H), 3.08 – 3.02 (m, 2H). **^{13}C NMR** (151 MHz, $CDCl_3$) δ 167.92, 150.81, 148.99, 148.07, 145.61, 132.90, 131.27, 128.08, 122.16, 120.36, 120.01, 117.48, 108.32, 104.47, 102.84, 101.77, 56.03, 53.19, 28.57. **HRMS** Accurate mass (ES+): Found 322.10777 (+1.21 ppm), $C_{19}H_{16}NO_4$ (M^+) requires 322.10738.

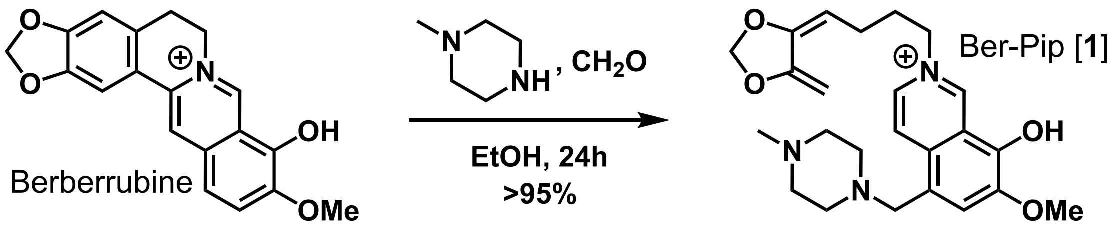

Ber-Pip [1]: 9-hydroxy-10-methoxy-12-((4-methylpiperazin-1-yl)methyl)-5,6-dihydro-[1,3]dioxolo[4,5-g]isoquinolino[3,2-a]isoquinolin-7-ium chloride.[3] To a solution of berberrubine (24 mg, 0.067 mmol, 1 eq.) in anhydrous EtOH (1 mL) was added N-methylpiperazine (0.037 mL, 0.335 mmol, 5 eq.) and formaldehyde (37% aq., 0.035 mL, 0.335 mmol, 5 eq.). The solution stirred at room temperature for 24 hours and was then concentrated *in vacuo* and purified by silica column chromatography (0-5% MeOH/DCM) to afford the title compound as a yellow powder (30.5 mg, >95% yield). **^{1}H NMR** (600 MHz, $CDCl_3$) δ 9.21 (s, 1H), 8.03 (s, 1H), 7.21 (s, 1H), 7.18 (s, 1H), 6.70 (s, 1H), 6.02 (s, 2H), 4.38 (t, *J* = 5.9 Hz, 2H), 3.86 (s, 3H), 3.65 (s, 2H), 3.04 (t, *J* = 6.0 Hz, 2H), 2.52 (s, 8H), 2.29 (s, 3H). **^{13}C NMR** (151 MHz, $CDCl_3$) δ 167.15, 149.90, 149.15, 148.21, 145.75, 132.96, 129.71, 128.33, 123.82, 122.55, 120.47, 115.07, 110.00, 108.45, 104.71, 101.87, 64.39, 59.75, 56.33, 55.19, 53.30, 52.68, 45.81, 28.64, 25.49. **HRMS** Accurate mass (ES+): Found 434.20693 (-1.17 ppm), $C_{25}H_{28}N_3O_4$ (M^+) requires 434.20743.

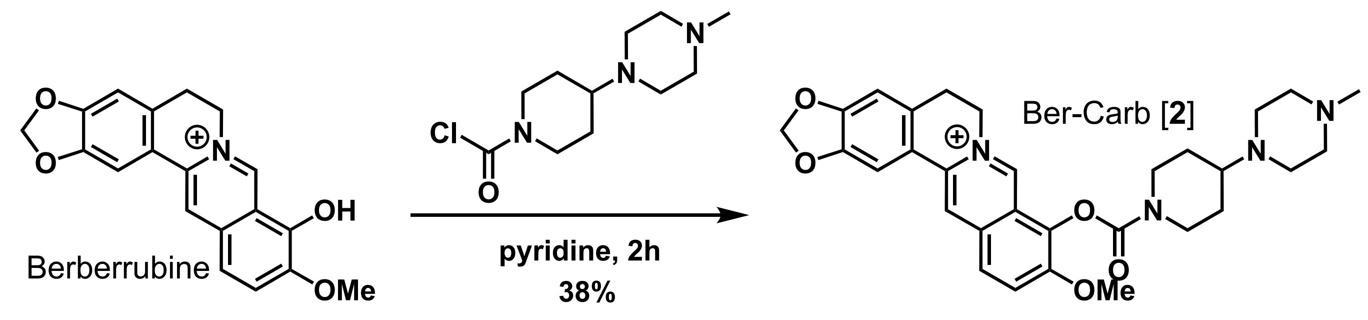

Ber-Carb [2]: 10-methoxy-9-((4-(4-methylpiperazin-1-yl)piperidine-1-carbonyl)oxy)-5,6-dihydro-[1,3]dioxolo[4,5-g]isoquinolino[3,2-a]isoquinolin-7-ium chloride formate.[4] Berberrubine (96.4 mg, 0.300 mmol, 1 eq.) was dissolved in pyridine (1 mL) and added in one portion to 4-(4-methylpiperazin-1-yl)piperidine-1-carbonyl chloride (369 mg, 1.50 mmol, 5 eq.)**.** The solution was stirred at room temperature for 2 hours, then was concentrated *in vacuo* and purified by silica column chromatography (0-5% MeOH/DCM) to yield the title compound as a yellow solid (64.6 mg, 38% yield) **^{1}H NMR** (600 MHz, D_2O) δ 9.24 (s, 1H), 8.41 (s, 1H), 8.15 (s, 1H), 7.84 (d, *J* = 19.0 Hz, 2H), 7.14 (s, 1H), 6.80 (s, 1H), 6.00 (s, 2H), 4.65 (s, 2H), 4.40 (s, 1H), 4.11 (d, *J* = 12.9 Hz, 1H), 3.92 (s, 3H), 3.68 – 3.52 (m, 2H), 3.22 – 3.16 (m, 1H), 3.09 – 2.97 (m, 4H), 2.62 (br s, 4H), 2.27 (s, 4H), 2.05 (br s, 2H), 1.56 (d, *J* = 8.5 Hz, 1H), 1.47 (d, *J* = 10.4 Hz, 1H). **^{13}C NMR** (151 MHz, D_2O) δ 171.02, 153.34, 150.46, 150.15, 147.64, 142.69, 138.04, 133.70, 132.89, 130.14, 126.43, 125.25, 121.36, 119.60, 108.28, 104.98, 102.28, 60.58, 56.89, 55.97, 53.54, 47.84, 27.57, 27.23, 26.40. **HRMS** Accurate mass (ES+): Found 531.26012 (-0.14 ppm), $C_{30}H_{35}N_4O_5$ (M^+) requires 531.26020.

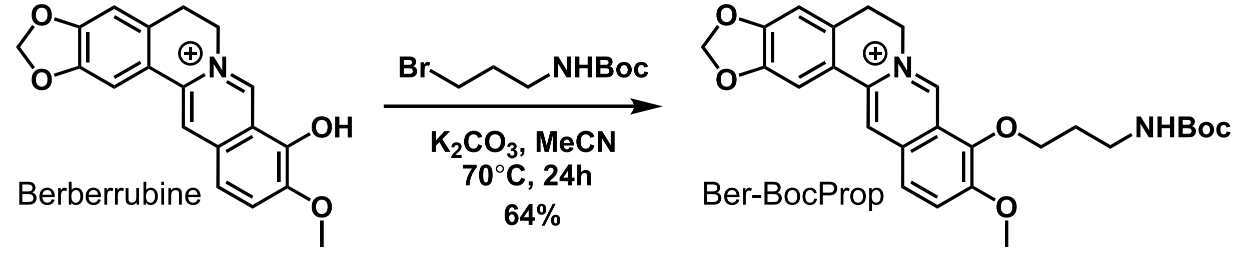

9-(3-((tert-butoxycarbonyl)amino)propoxy)-10-methoxy-5,6-dihydro-[1,3]dioxolo[4,5-g]isoquinolino[3,2-a]isoquinolin-7-ium chloride. To a solution of berberrubine (50 mg, 0.134 mmol, 1 eq.) and K_2CO_3 (18.6 mg, 0.403 mmol, 3 eq.) in acetonitrile (1 mL) preheated to 70°C was added tert-butyl (3-bromopropyl)carbamate (64 mg, 0.269 mmol, 2 eq.). Additional acetonitrile (0.5 mL) was added, the vessel was sealed, and the reaction continued to stir at 70°C for 24 hours. The crude reaction mixture was cooled to RT and purified by silica column chromatography (0-10% MeOH/DCM), yielding the title compound as a yellow powder (45.9 mg, 64%) **^{1}H NMR** (500 MHz, DMSO-d_6) δ 9.83 (s, 1H), 8.93 (s, 1H), 8.19 (d, *J* = 9.2 Hz, 1H), 7.97 (d, *J* = 9.0 Hz, 1H), 7.79 (s, 1H), 7.09 (s, 1H), 6.97 (t, *J* = 5.7 Hz, 1H), 6.16 (s, 2H), 5.00 – 4.89 (m, 2H), 4.28 (t, *J* = 6.0 Hz, 2H), 4.04 (s, 3H), 3.24 – 3.15 (m, 4H), 1.94 (p, *J* = 5.9, 5.5 Hz, 2H), 1.34 (s, 9H). **^{13}C NMR** (151 MHz, DMSO-d_6) δ 155.87, 150.28, 149.80, 147.67, 145.43, 142.73, 137.37, 132.98, 130.60, 126.63, 123.34, 121.53, 120.43, 120.16, 108.43, 105.43, 102.10, 77.65, 71.80, 57.06, 55.30, 54.95, 36.68, 30.28, 28.25, 26.34. **HRMS** Accurate mass (ES+): Found 479.21776 (-0.14 ppm), $C_{27}H_{31}N_2O_6$ (M^+) requires 479.21766.

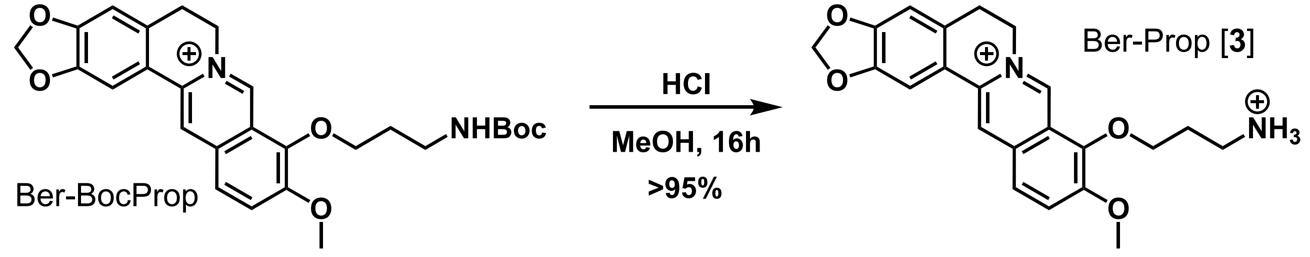

Ber-Prop [3]: 9-(3-ammoniopropoxy)-10-methoxy-5,6-dihydro-[1,3]dioxolo[4,5-g]isoquinolino[3,2-a]isoquinolin-7-ium dichloride. Carbamate **Ber-BocProp** (10 mg, 0.019 mmol, 1 eq.) was dissolved in MeOH (4 mL) and HCl (12M, 2.0 mL, 24 mmol) was added. Reaction was stirred at room temperature for 24 hours, then solvent was removed *in vacuo* to afford the pure HCl salt product as a yellow powder (8.5 mg, >95% yield). ^{1}**H NMR** (600 MHz, DMSO-d_6) δ 9.98 (s, 1H), 8.94 (s, 1H), 8.24 – 8.17 (m, 4H), 7.98 (d, *J* = 9.1 Hz, 1H), 7.79 (s, 1H), 7.08 (s, 1H), 6.16 (s, 2H), 5.01 (t, *J* = 6.2 Hz, 2H), 4.37 (t, *J* = 5.8 Hz, 2H), 4.07 (s, 3H), 3.22 – 3.17 (m, 2H), 3.10 (q, *J* = 6.7, 6.3 Hz, 2H), 2.18 (p, *J* = 6.3 Hz, 2H). ^{13}**C NMR** (151 MHz, DMSO) δ 150.66, 150.32, 148.18, 145.96, 143.14, 137.97, 133.44, 131.21, 127.11, 123.97, 121.93, 120.93, 120.67, 108.93, 105.91, 102.58, 72.04, 57.60, 55.60, 36.64, 28.30, 26.80. **HRMS** Accurate mass (ES-): Found 379.16512 (-0.31 ppm), $C_{22}H_{23}N_2O_4$ (M^{2+} -H^{+}) requires 379.16523.

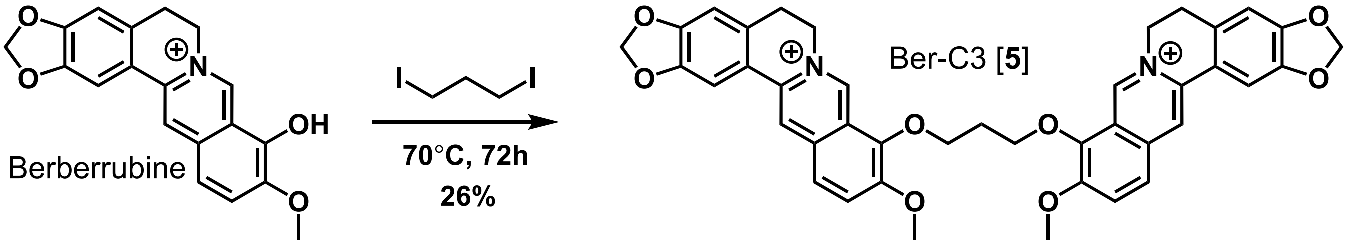

Ber-C3 [5]: 9,9'-(propane-1,4-diylbis(oxy))bis(10-methoxy-5,6-dihydro-[1,3]dioxolo[4,5-g]isoquinolino[3,2-a]isoquinolin-7-ium) dichloride. Following general procedure A, diiodide (16.6 mg. 0.056 mmol) yielded the title compound as a yellow solid (11.2 mg, 26% yield). ^{1}**H NMR** (600 MHz, DMSO-d_6) δ 9.85 (s, 2H), 8.97 (s, 2H), 8.22 (d, *J* = 9.2 Hz, 2H), 8.01 (d, *J* = 9.1 Hz, 2H), 7.81 (s, 2H), 7.11 (s, 2H), 6.19 (s, 4H), 4.97 – 4.92 (m, 4H), 4.59 (t, *J* = 6.4 Hz, 4H), 4.04 (s, 6H), 3.24 – 3.19 (m, 4H), 2.57 – 2.52 (m, 2H). ^{13}**C NMR** (151 MHz, DMSO) δ 150.86, 150.36, 148.21, 145.77, 143.24, 138.02, 133.54, 131.17, 127.24, 123.98, 122.05, 120.90, 120.74, 108.92, 105.93, 102.61, 72.15, 57.60, 55.76, 30.92, 26.80. **HRMS** Accurate mass (ES+): Found 342.12269 (-1.01 ppm), $C_{41}H_{36}N_2O_8$ (M^{2+}) requires 342.12303.

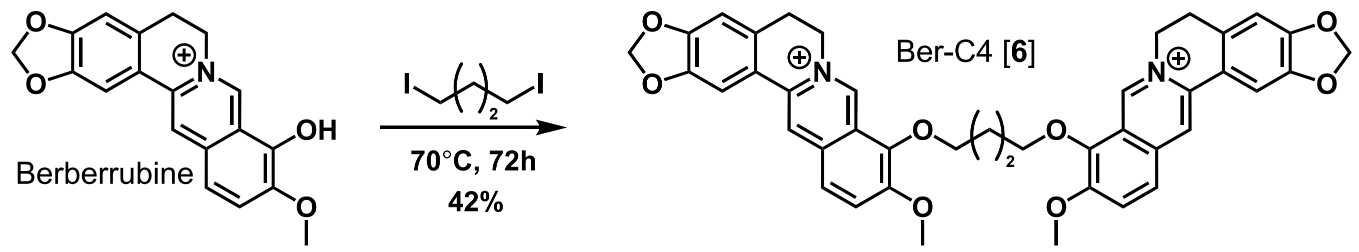

Ber-C4 [6]: 9,9'-(butane-1,4-diylbis(oxy))bis(10-methoxy-5,6-dihydro-[1,3]dioxolo[4,5-g]isoquinolino[3,2-a]isoquinolin-7-ium) dichloride. Following general procedure A, diiodide (17.4 mg. 0.056 mmol) yielded the title compound as a yellow solid (18.3 mg, 42% yield). ^{1}**H NMR** (500 MHz, DMSO-*d*$_6$) δ 9.82 (s, 2H), 8.96 (s, 2H), 8.21 (d, *J* = 7.9 Hz, 2H), 8.00 (d, *J* = 7.9 Hz, 2H), 7.81 (s, 2H), 7.10 (s, 2H), 6.18 (s, 4H), 4.96 (s, 4H), 4.41 (s, 4H), 4.05 (s, 6H), 3.20 (s, 4H), 2.14 (s, 4H). ^{13}**C NMR** (151 MHz, DMSO-*d*$_6$) δ 150.90, 150.32, 148.18, 145.76, 143.17, 137.97, 133.47, 131.16, 127.10, 123.92, 122.11, 120.91, 120.73, 108.92, 105.90, 102.61, 76.98, 74.41, 57.51, 55.78, 26.58. **HRMS** Accurate mass (ES+): Found 349.13082 (-0.11 ppm), $C_{42}H_{38}N_2O_8$ (M^{2+}) requires 349.13086.

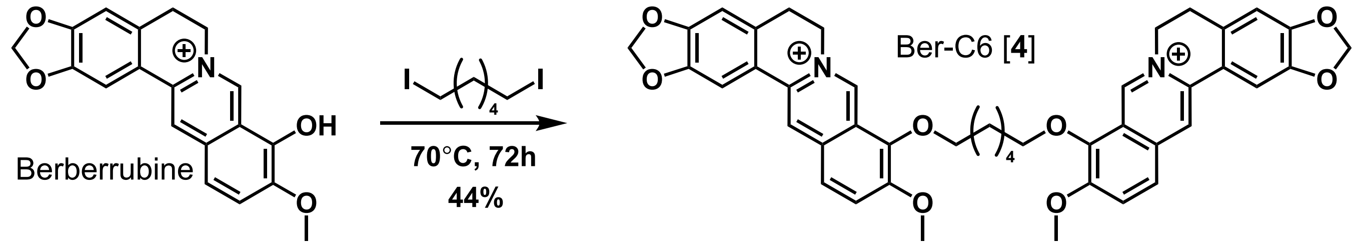

Ber-C6 [4]: 9,9'-(hexane-1,4-diylbis(oxy))bis(10-methoxy-5,6-dihydro-[1,3]dioxolo[4,5-g]isoquinolino[3,2-a]isoquinolin-7-ium) dichloride. Following general procedure A, diiodide (38.0 mg. 0.112 mmol) yielded the title compound as a yellow solid (39.3 mg, 44% yield). ^{1}**H NMR** (600 MHz, DMSO-*d*$_6$) δ 9.75 (s, 2H), 8.93 (s, 2H), 8.20 (d, *J* = 9.2 Hz, 2H), 7.99 (d, *J* = 9.1 Hz, 2H), 7.78 (s, 2H), 7.09 (s, 2H), 6.17 (s, 4H), 4.94 (t, *J* = 6.1 Hz, 4H), 4.31 (t, *J* = 6.7 Hz, 4H), 4.04 (s, 6H), 3.19 (t, *J* = 6.1 Hz, 4H), 1.94 (p, *J* = 6.2 Hz, 4H), 1.60 (p, *J* = 4.0 Hz, 4H). ^{13}**C NMR** (151 MHz, DMSO) δ 150.41, 149.84, 147.69, 145.20, 142.83, 137.47, 133.03, 130.64, 126.70, 123.34, 121.66, 120.41, 120.23, 108.40, 105.39, 102.11, 74.23, 57.03, 55.26, 29.48, 26.31, 25.13. **HRMS** Accurate mass (ES+): Found 363.14631 (-0.55 ppm), $C_{44}H_{42}N_2O_8$ (M^{2+}) requires 363.14651.

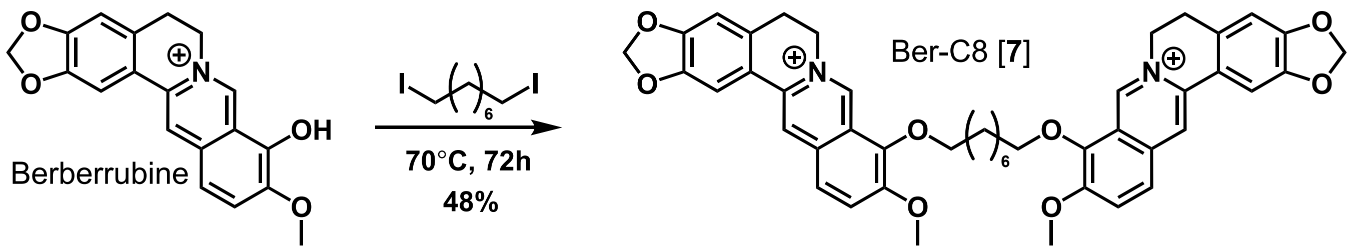

Ber-C8 [7]: 9,9'-(octane-1,8-diylbis(oxy))bis(10-methoxy-5,6-dihydro-[1,3]dioxolo[4,5-g]isoquinolino[3,2-a]isoquinolin-7-ium) dichloride. Following general procedure A, diiodide (20.5 mg. 0.056 mmol) yielded the title compound as a yellow solid (22.2 mg, 48% yield). ^{1}**H NMR** (600 MHz, DMSO-d_6) δ 9.75 (s, 2H), 8.94 (s, 2H), 8.20 (d, J = 9.2 Hz, 2H), 8.00 (d, J = 9.1 Hz, 2H), 7.79 (s, 2H), 7.09 (s, 2H), 6.18 (s, 4H), 4.98 – 4.93 (m, 4H), 4.30 (t, J = 6.6 Hz, 4H), 4.04 (s, 6H), 3.23 – 3.18 (m, 4H), 1.91 (dt, J = 14.3, 6.9 Hz, 4H), 1.55 – 1.49 (m, 4H), 1.44 (s, 4H). ^{13}**C NMR** (151 MHz, DMSO) δ 150.88, 150.30, 148.16, 145.71, 143.34, 137.94, 133.51, 131.13, 127.19, 123.79, 122.14, 120.91, 120.71, 108.88, 105.89, 102.58, 74.76, 57.54, 55.78, 29.99, 29.33, 26.80, 25.76. **HRMS** Accurate mass (ES+): Found 377.16182 (-0.90 ppm), $C_{46}H_{46}N_2O_8$ (M^{2+}) requires 377.16216.

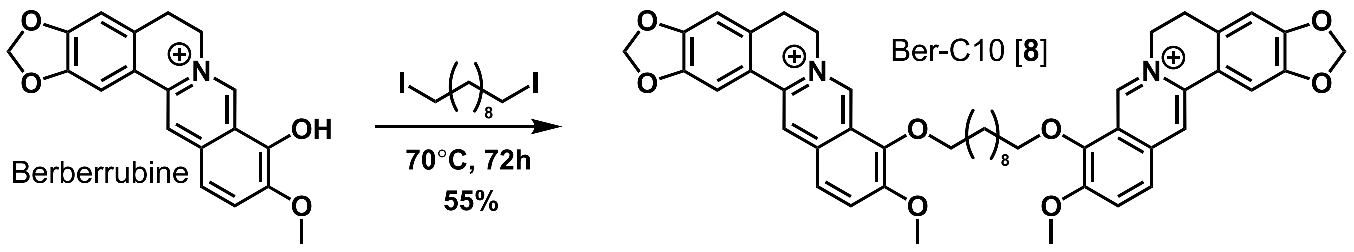

Ber-C10 [8]: 9,9'-(decane-1,4-diylbis(oxy))bis(10-methoxy-5,6-dihydro-[1,3]dioxolo[4,5-g]isoquinolino[3,2-a]isoquinolin-7-ium) dichloride. Following general procedure A, diiodide (23.0 mg. 0.058 mmol) yielded the title compound as a yellow solid (27.3 mg, 55% yield). ^{1}**H NMR** (600 MHz, DMSO-d_6) δ 9.75 (s, 2H), 8.95 (s, 2H), 8.20 (d, J = 9.1 Hz, 2H), 8.00 (d, J = 9.0 Hz, 2H), 7.80 (s, 2H), 7.09 (s, 2H), 6.18 (s, 4H), 4.98 – 4.93 (m, 4H), 4.29 (t, J = 6.7 Hz, 4H), 4.05 (s, 6H), 3.21 (d, J = 11.3 Hz, 4H), 1.89 (dt, J = 14.4, 6.9 Hz, 4H), 1.51 – 1.46 (m, 4H), 1.41 – 1.22 (m, 22H). ^{13}**C NMR** (151 MHz, DMSO) δ 150.89, 150.30, 148.17, 145.74, 143.35, 137.94, 133.51, 131.15, 127.18, 123.78, 122.14, 120.92, 120.70, 108.89, 105.90, 102.58, 74.74, 57.54, 55.79, 29.99, 29.54, 29.36, 26.81, 25.78. **HRMS** Accurate mass (ES+): Found 391.1773 (-1.31 ppm), $C_{48}H_{90}N_2O_8$ (M^{2+}) requires 391.17781.

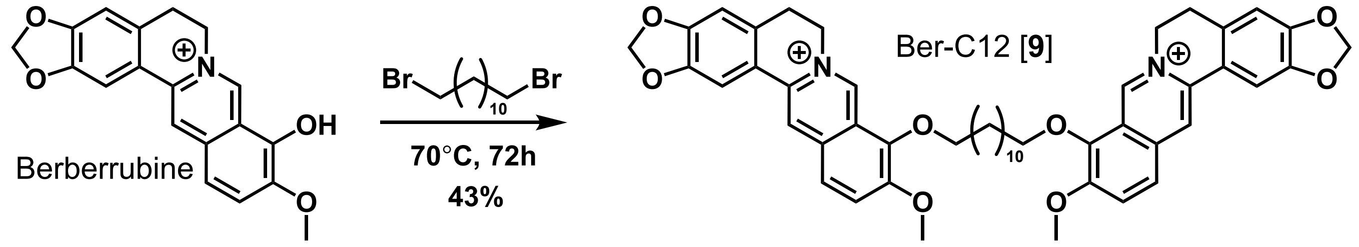

Ber-C12 [9]: 9,9'-(dodecane-1,4-diylbis(oxy))bis(10-methoxy-5,6-dihydro-[1,3]dioxolo[4,5-g]isoquinolino[3,2-a]isoquinolin-7-ium) dichloride. Following general procedure B, dibromide (15.3 mg. 0.047 mmol) yielded the title compound as a yellow solid (17.8 mg, 43% yield). **^{1}H NMR** (600 MHz, DMSO-d_6) δ 9.75 (s, 2H), 8.94 (s, 2H), 8.20 (d, *J* = 9.1 Hz, 2H), 7.99 (d, *J* = 9.0 Hz, 2H), 7.80 (s, 2H), 7.09 (s, 2H), 6.18 (s, 4H), 4.99 – 4.91 (m, 4H), 4.28 (t, *J* = 6.5 Hz, 4H), 4.05 (s, 6H), 3.23 – 3.18 (m, 4H), 1.93 – 1.82 (m, 4H), 1.52 – 1.43 (m, 4H), 1.40 – 1.21 (m, 22H). **^{13}C NMR** (151 MHz, DMSO) δ 150.89, 150.31, 148.18, 145.75, 143.35, 137.94, 133.51, 131.15, 127.19, 123.78, 122.15, 120.93, 120.72, 108.90, 105.90, 102.58, 74.74, 57.53, 55.79, 29.99, 29.57, 29.50, 29.37, 26.82, 25.77. **HRMS** Accurate mass (ES+): Found 405.19332 (-0.33 ppm), $C_{50}H_{54}N_2O_8$ (M^{2+}) requires 405.19346.

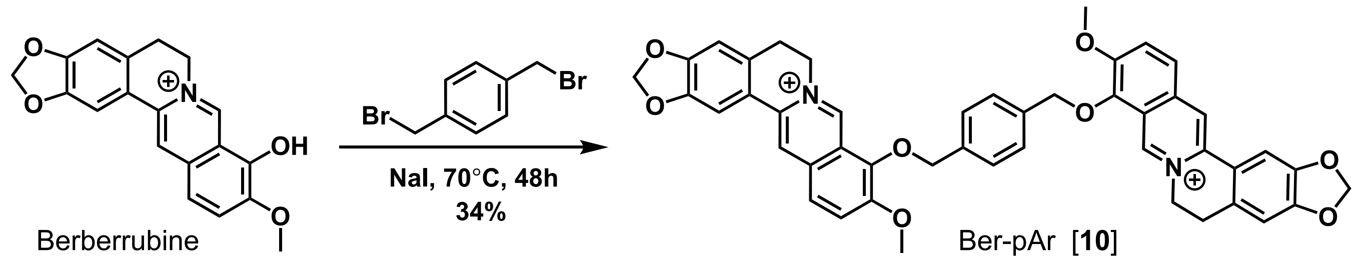

Ber-pAr [10]: 9,9'-((1,4-phenylenebis(methylene))bis(oxy))bis(10-methoxy-5,6-dihydro-[1,3]dioxolo[4,5-g]isoquinolino[3,2-a]isoquinolin-7-ium) dichloride. Following general procedure B, dibromide (29.2 mg. 0.111 mmol) yielded the title compound as a yellow solid (34.0 mg, 34% yield). **^{1}H NMR** (600 MHz, DMSO-d_6) δ 9.70 (s, 2H), 8.91 (s, 2H), 8.21 (d, *J* = 9.2 Hz, 2H), 8.00 (d, *J* = 9.0 Hz, 2H), 7.75 (s, 2H), 7.60 (s, 4H), 7.05 (s, 2H), 6.16 (s, 4H), 5.34 (s, 4H), 4.91 (t, *J* = 6.2 Hz, 4H), 4.07 (s, 6H), 3.22 – 3.14 (t, *J* = 6.2 Hz, 4H). **^{13}C NMR** (151 MHz, DMSO) δ 150.67, 149.82, 147.66, 145.16, 141.79, 137.38, 136.58, 132.87, 130.59, 128.85, 126.52, 123.79, 121.82, 120.33, 120.17, 108.35, 105.35, 102.11, 75.02, 57.03, 55.25, 26.30. **HRMS** Accurate mass (ES+): Found 373.13073 (-0.35 ppm), $C_{46}H_{38}N_2O_8$ (M^{2+}) requires 373.13086.

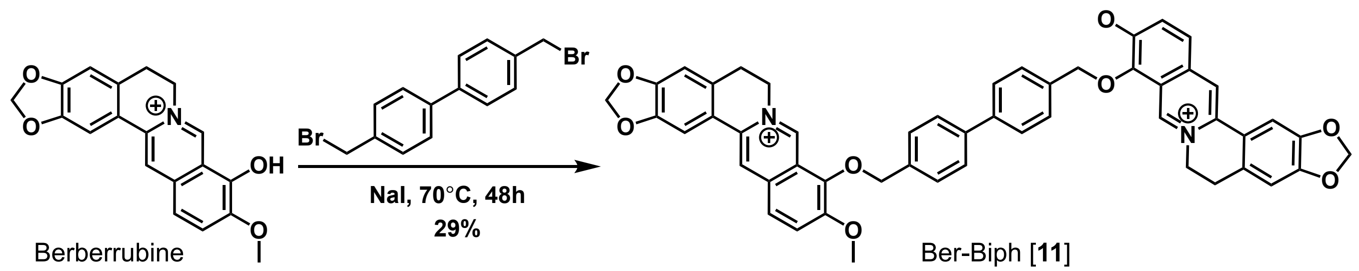

9,9'-Ber-Biph [11]: (([1,1'-biphenyl]-4,4'-diylbis(methylene))bis(oxy))bis(10-methoxy-5,6-dihydro-[1,3]dioxolo[4,5-g]isoquinolino[3,2-a]isoquinolin-7-ium) dichloride. Following general procedure B, dibromide (38.2 mg, 0.112 mmol) yielded the title compound as a yellow solid (28.5 mg, 29% yield). **^{1}H NMR** (600 MHz, DMSO-d_6) δ 9.80 (s, 2H), 8.95 (s, 2H), 8.24 (d, J = 9.2 Hz, 2H), 8.02 (d, J = 9.1 Hz, 2H), 7.80 (s, 2H), 7.74 (d, J = 8.3 Hz, 4H), 7.70 (d, J = 8.4 Hz, 4H), 7.10 (s, 2H), 6.18 (s, 4H), 5.41 (s, 4H), 4.94 (t, J = 6.4 Hz, 4H), 4.11 (s, 6H), 3.20 (t, J = 6.4, 4H). **^{13}C NMR** (151 MHz, DMSO-d_6) δ 150.69, 149.88, 147.72, 145.35, 142.06, 139.62, 137.48, 135.91, 132.97, 130.68, 129.65, 129.38, 126.63, 123.79, 121.80, 120.42, 120.28, 108.44, 105.44, 102.12, 75.05, 57.09, 55.34, 26.35. **HRMS** Accurate mass (ES+): Found 411.14652 (+0.02 ppm), $C_{52}H_{42}N_2O_8$ (M^{2+}) requires 411.14651.

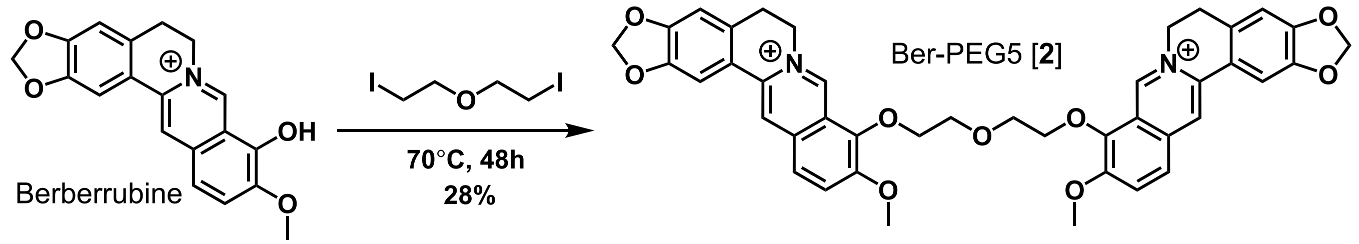

Ber-PEG5 [12]: 9,9'-((oxybis(ethane-2,1-diyl))bis(oxy))bis(10-methoxy-5,6-dihydro-[1,3]dioxolo[4,5-g]isoquinolino[3,2-a]isoquinolin-7-ium) dichloride. Following general procedure A, diiodide (15.0 mg. 0.046 mmol) yielded the title compound as a yellow solid (12.3 mg, 28% yield). **^{1}H NMR** (600 MHz, DMSO-d_6) δ 9.54 (s, 2H), 8.75 (s, 2H), 8.09 (d, *J* = 9.2 Hz, 2H), 7.93 – 7.85 (m, 2H), 7.39 (s, 2H), 7.04 (s, 2H), 6.13 (s, 4H), 4.88 (t, *J* = 5.7 Hz, 4H), 4.30 – 4.23 (m, 4H), 3.92 (s, 6H), 3.88 – 3.84 (m, 4H), 3.18 – 3.11 (m, 4H). **^{13}C NMR** (151 MHz, DMSO) δ 150.14, 149.70, 147.50, 145.08, 142.58, 137.04, 132.77, 130.19, 126.10, 123.56, 121.82, 120.11, 119.95, 108.22, 105.04, 102.14, 72.83, 69.48, 56.79, 55.06, 26.16. **HRMS** Accurate mass (ES+): Found 357.12815 (-0.47 ppm), $C_{42}H_{38}N_2O_9$ (M^{2+}) requires 357.12832.

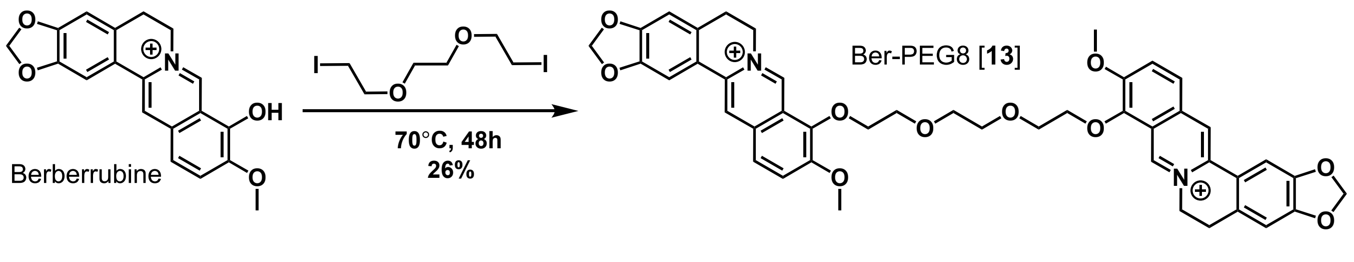

Ber-PEG8 [13]: 9,9'-(((ethane-1,2-diylbis(oxy))bis(ethane-2,1-diyl))bis(oxy))bis(10-methoxy-5,6-dihydro-[1,3]dioxolo[4,5-g]isoquinolino[3,2-a]isoquinolin-7-ium) dichloride. Following general procedure A, diiodide (17.0 mg. 0.047 mmol) yielded the title compound as a yellow solid (12.1 mg, 26% yield). ^{1}**H NMR** (600 MHz, DMSO-d_6) δ 9.69 (s, 2H), 8.86 (s, 2H), 8.11 (d, J = 9.2 Hz, 2H), 7.92 (d, J = 9.1 Hz, 2H), 7.68 (s, 2H), 7.05 (s, 2H), 6.15 (s, 4H), 4.92 (t, J = 6.1 Hz, 4H), 4.35 – 4.31 (m, 4H), 3.95 (s, 6H), 3.83 – 3.79 (m, 4H), 3.67 (s, 4H), 3.22 – 3.17 (m, 4H). ^{13}**C NMR** (151 MHz, DMSO) δ 150.77, 150.24, 148.08, 145.70, 142.80, 137.79, 133.28, 130.96, 126.79, 123.98, 122.10, 120.79, 120.55, 108.78, 105.73, 102.60, 73.66, 70.13, 69.90, 57.34, 55.89, 26.78. **HRMS** Accurate mass (ES+): Found 379.1414 (-0.07 ppm), $C_{44}H_{42}N_2O_{10}$ (M^{2+}) requires 379.14142.

Supplementary References

1. Sobel, M.L.; McKay, G.A.; Poole, K. Contribution of the MexXY Multidrug Transporter to Aminoglycoside Resistance in *Pseudomonas aeruginosa* Clinical Isolates. *AAC* 2003, **47**(10), 3202-3207. https://doi.org/10.1128/AAC.47.10.3202-3207.2003.
2. Chen, W. H.; Pang, J. Y.; Qin, Y.; Peng, Q.; Cai, Z.; Jiang, Z.H. Synthesis of Linked Berberine Dimers and Their Remarkably Enhanced DNA-Binding Affinities. *Bioorg Med Chem Lett* 2005, **15**(10), 2689–2692. https://doi.org/10.1016/j.bmcl.2004.10.098.
3. Li, R.; Wu, J.; He, Y.; Hai, L.; Wu, Y. Synthesis and in Vitro Evaluation of 12-(Substituted Aminomethyl) Berberrubine Derivatives as Anti-Diabetics. *Bioorg Med Chem Lett* 2014, **24**(7), 1762–1765. https://doi.org/10.1016/j.bmcl.2014.02.032.
4. Huang, M.Y.; Lin, J.; Huang, Z.J.; Xu, H.G.; Hong, J.; Sun, P.-H.; Guo, J.L.; Chen, W.M. Design, Synthesis and Anti-Inflammatory Effects of Novel 9-O-Substituted-Berberine Derivatives. *Med Chem Commun* 2016, **7**(4), 658–666. https://doi.org/10.1039/C5MD00577A.

Appendix

The following pages show ^{1}H NMR (*top*) and ^{13}C NMR (*bottom*) spectra for all synthesized intermediates and berberine analogs. All spectra were taken in DMSO-d_6 with the exception of berberrubine ($CDCl_3$) and Ber-Carb [**2**] (D_2O).

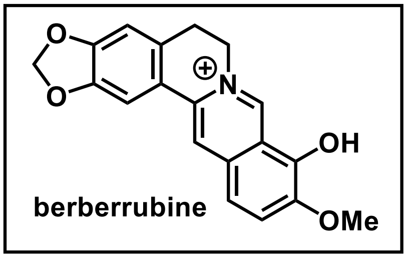
O
O
N
OH
OMe
berberrubine

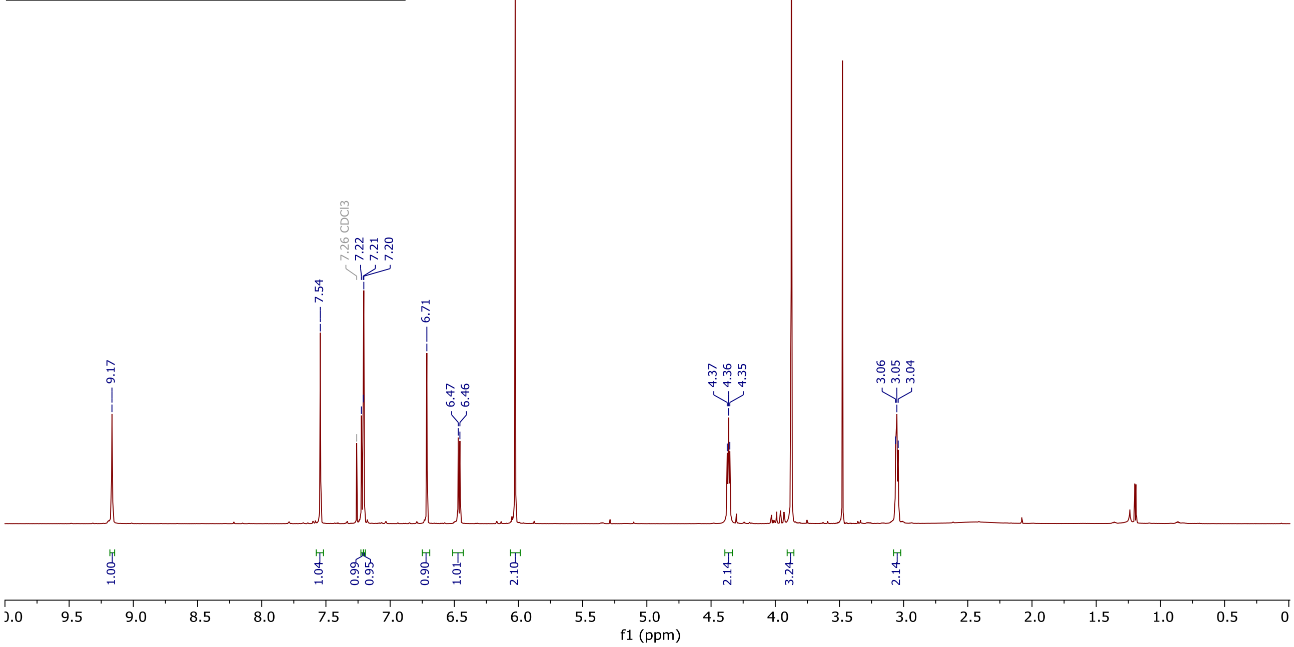
3.88
6.03
7.26 CDCl3
7.22
7.21
7.20
7.54
6.71
9.17
6.47
6.46
4.37
4.36
4.35
3.06
3.05
3.04
1.00
1.04
0.99
0.95
0.90
1.01
2.10
2.14
3.24
2.14
f1 (ppm)

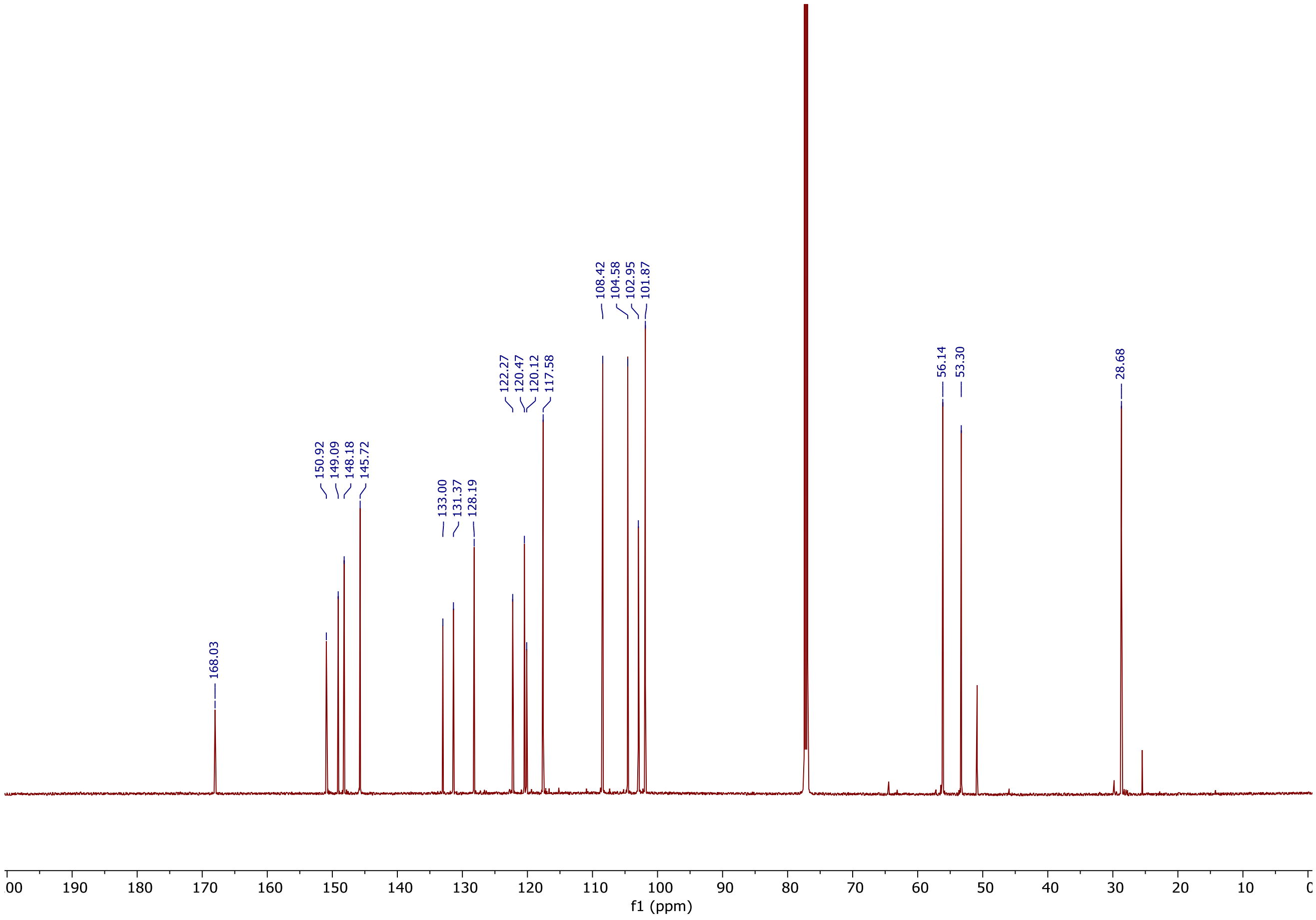
108.42
104.58
102.95
101.87
122.27
120.47
120.12
117.58
56.14
53.30
28.68
150.92
149.09
148.18
145.72
133.00
131.37
128.19
168.03
f1 (ppm)

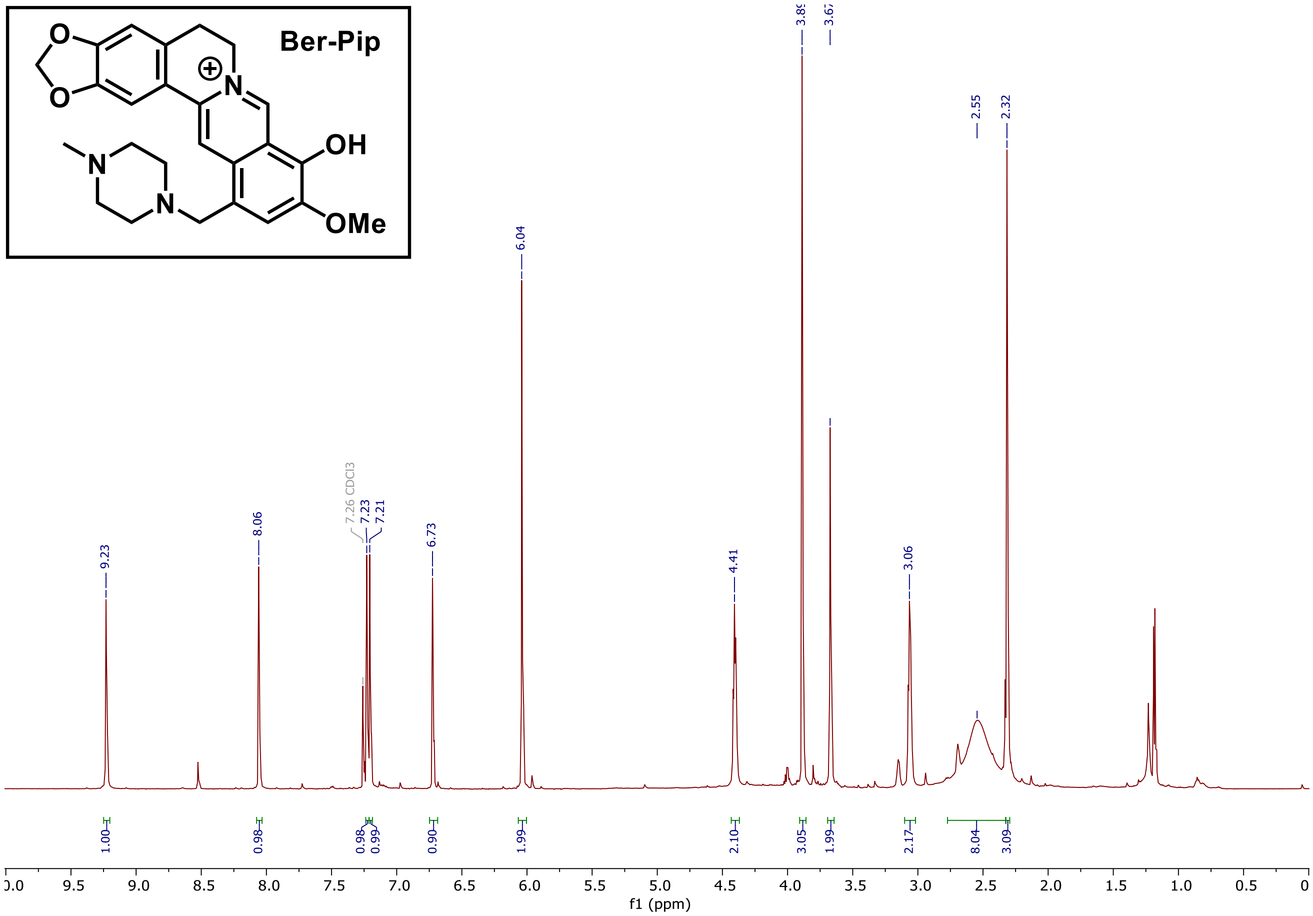
Ber-Pip
OH
OMe
9.23
8.06
7.26 CDCl3
7.23
7.21
6.73
6.04
4.41
3.89
3.67
3.06
2.55
2.32
1.00
0.98
0.98
0.99
0.90
1.99
2.10
3.05
1.99
2.17
8.04
3.09
f1 (ppm)

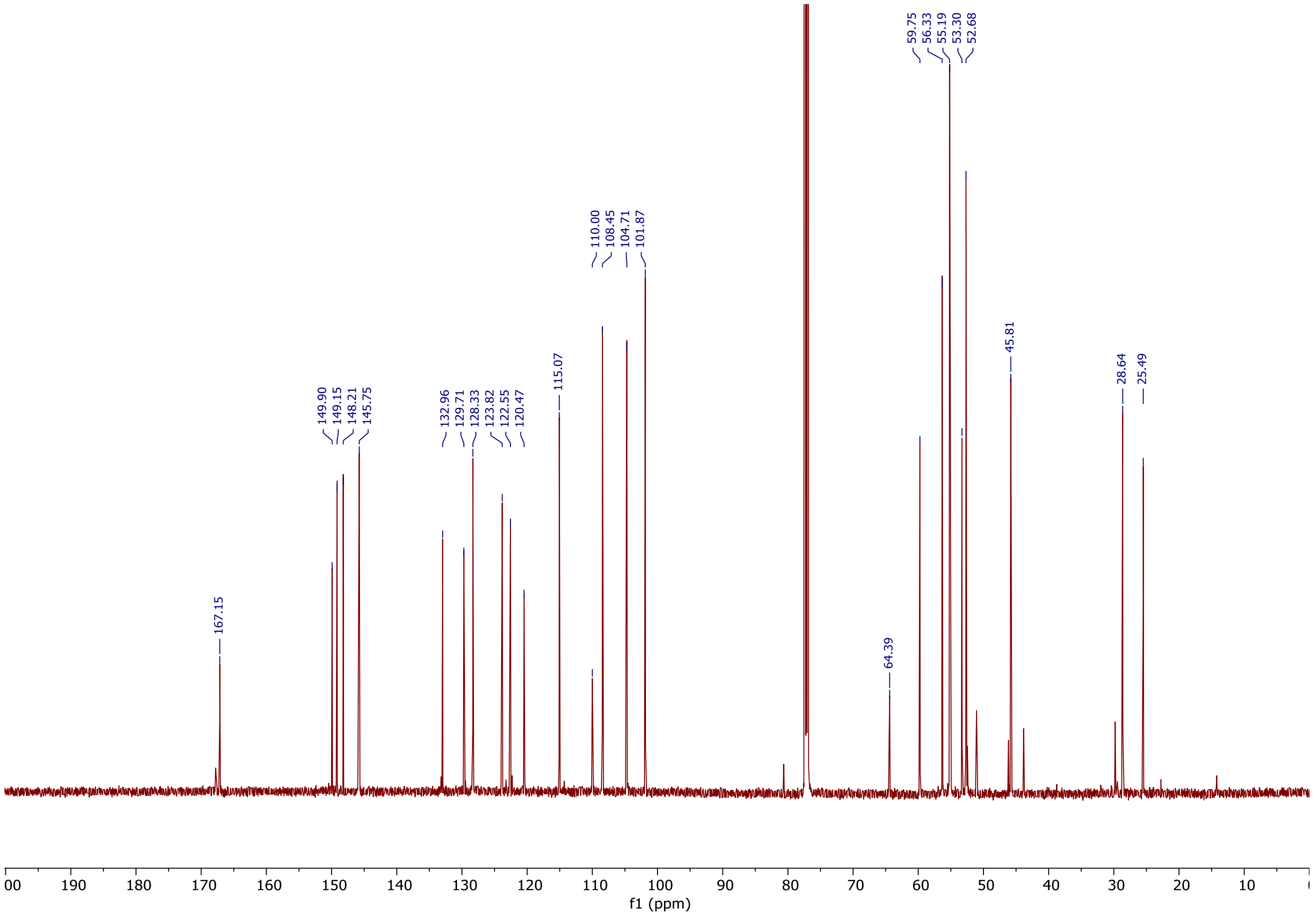
167.15
149.90
149.15
148.21
145.75
132.96
129.71
128.33
123.82
122.55
120.47
115.07
110.00
108.45
104.71
101.87
64.39
59.75
56.33
55.19
53.30
52.68
45.81
28.64
25.49
f1 (ppm)

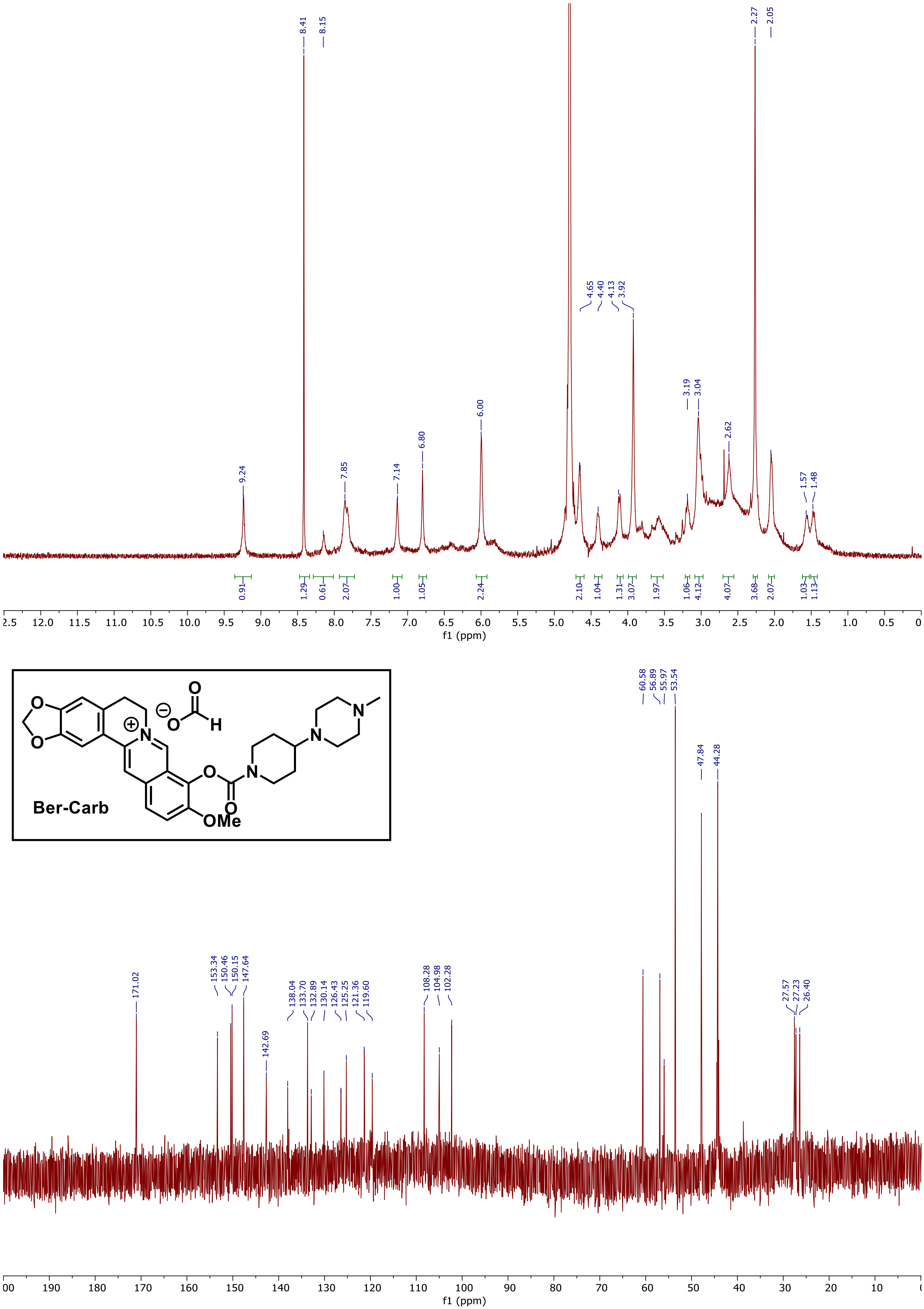
9.24
8.41
8.15
7.85
7.14
6.80
6.00
4.65
4.40
4.13
3.92
3.19
3.04
2.62
2.27
2.05
1.57
1.48
0.91
1.29
0.61
2.07
1.00
1.05
2.24
2.10
1.04
1.31
3.07
1.97
1.06
4.12
4.07
3.68
2.07
1.03
1.13
f1 (ppm)
Ber-Carb
OMe
171.02
153.34
150.46
150.15
147.64
142.69
138.04
133.70
132.89
130.14
126.43
125.25
121.36
119.60
108.28
104.98
102.28
60.58
56.89
55.97
53.54
47.84
44.28
27.57
27.23
26.40
f1 (ppm)

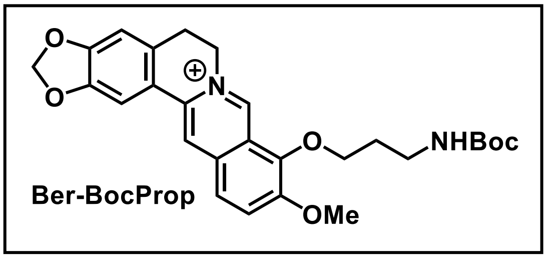
NHBoc
OMe
Ber-BocProp

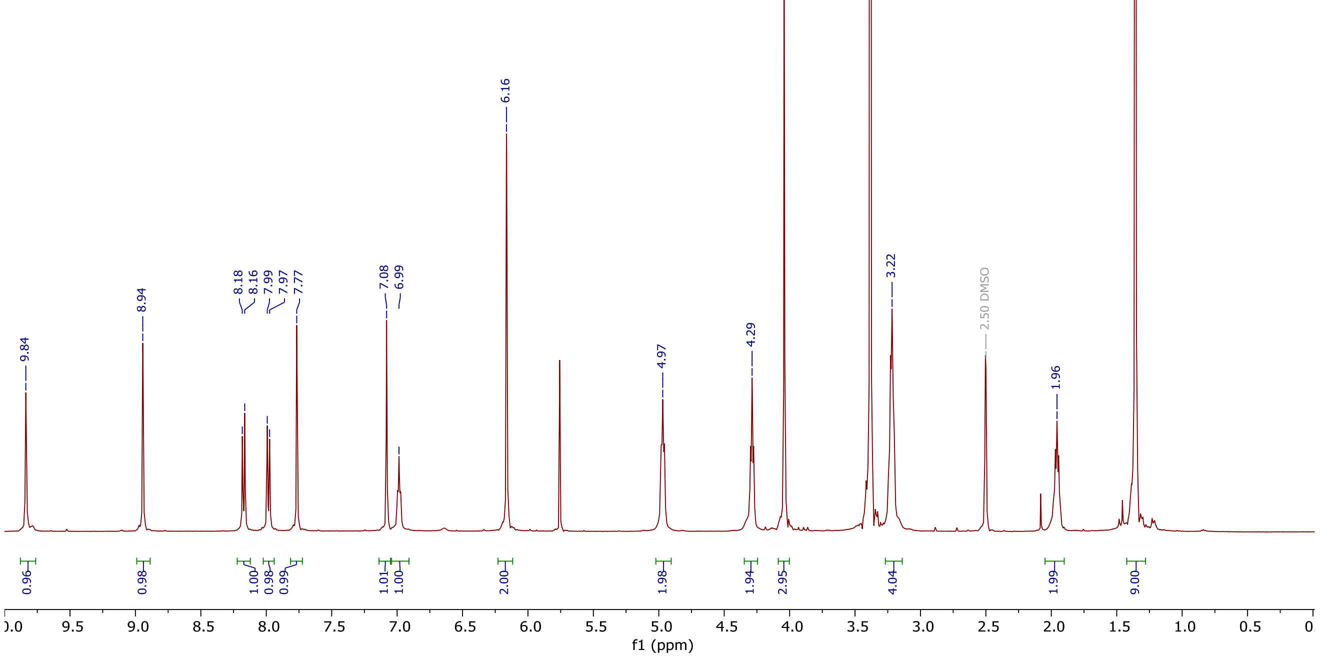
9.84
8.94
8.18
8.16
7.99
7.97
7.77
7.08
6.99
6.16
4.97
4.29
4.04
3.22
2.50 DMSO
1.96
0.96
0.98
1.00
0.98
0.99
1.01
1.00
2.00
1.98
1.94
2.95
4.04
1.99
9.00
f1 (ppm)

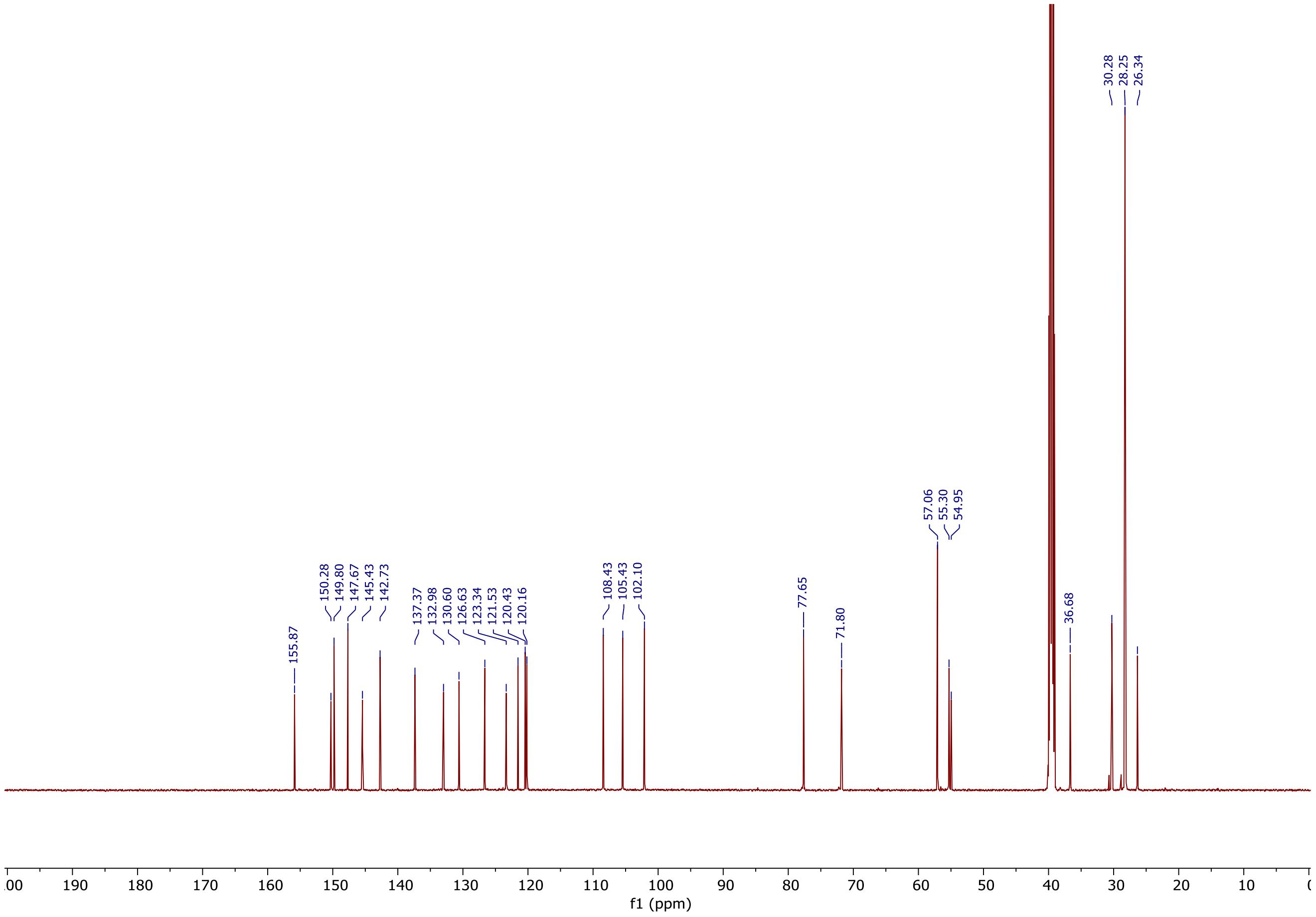
155.87
150.28
149.80
147.67
145.43
142.73
137.37
132.98
130.60
126.63
123.34
121.53
120.43
120.16
108.43
105.43
102.10
77.65
71.80
57.06
55.30
54.95
36.68
30.28
28.25
26.34
f1 (ppm)

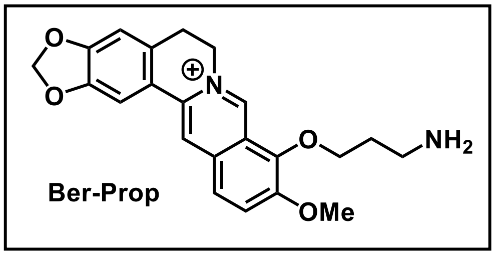
Ber-Prop
NH2
OMe

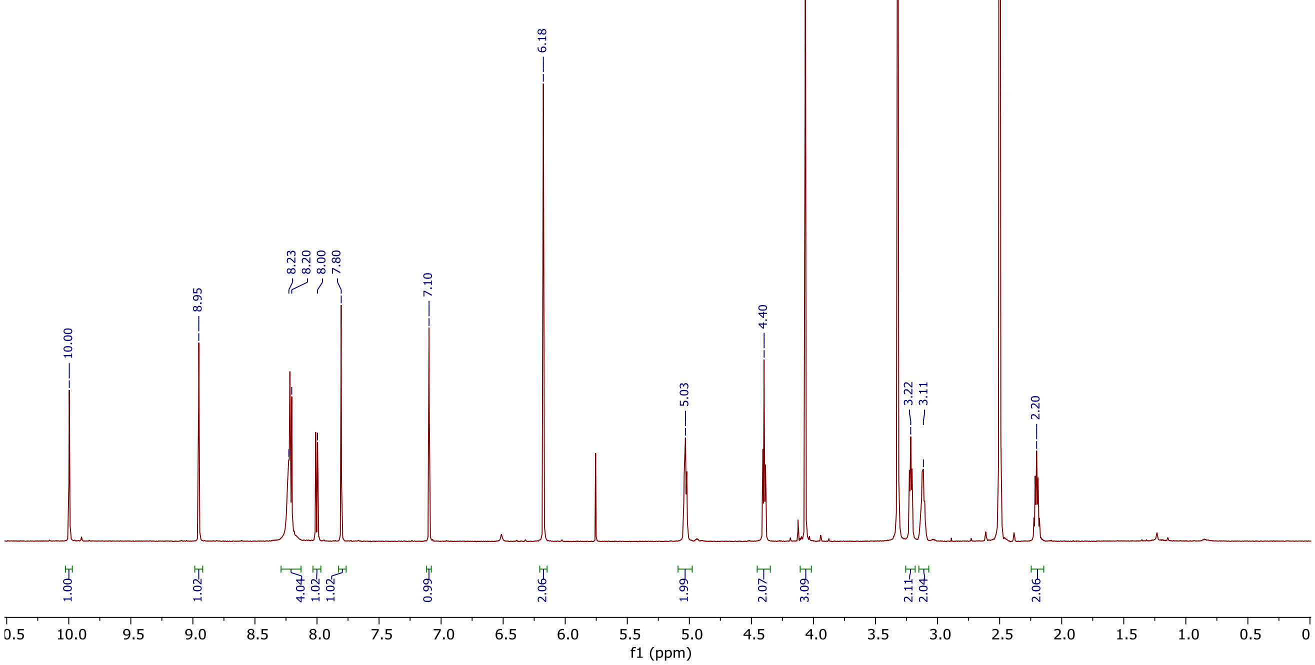
10.00
8.95
8.23
8.20
8.00
7.80
7.10
6.18
5.03
4.40
4.07
3.22
3.11
2.20
1.00
1.02
4.04
1.02
1.02
0.99
2.06
1.99
2.07
3.09
2.11
2.04
2.06
f1 (ppm)

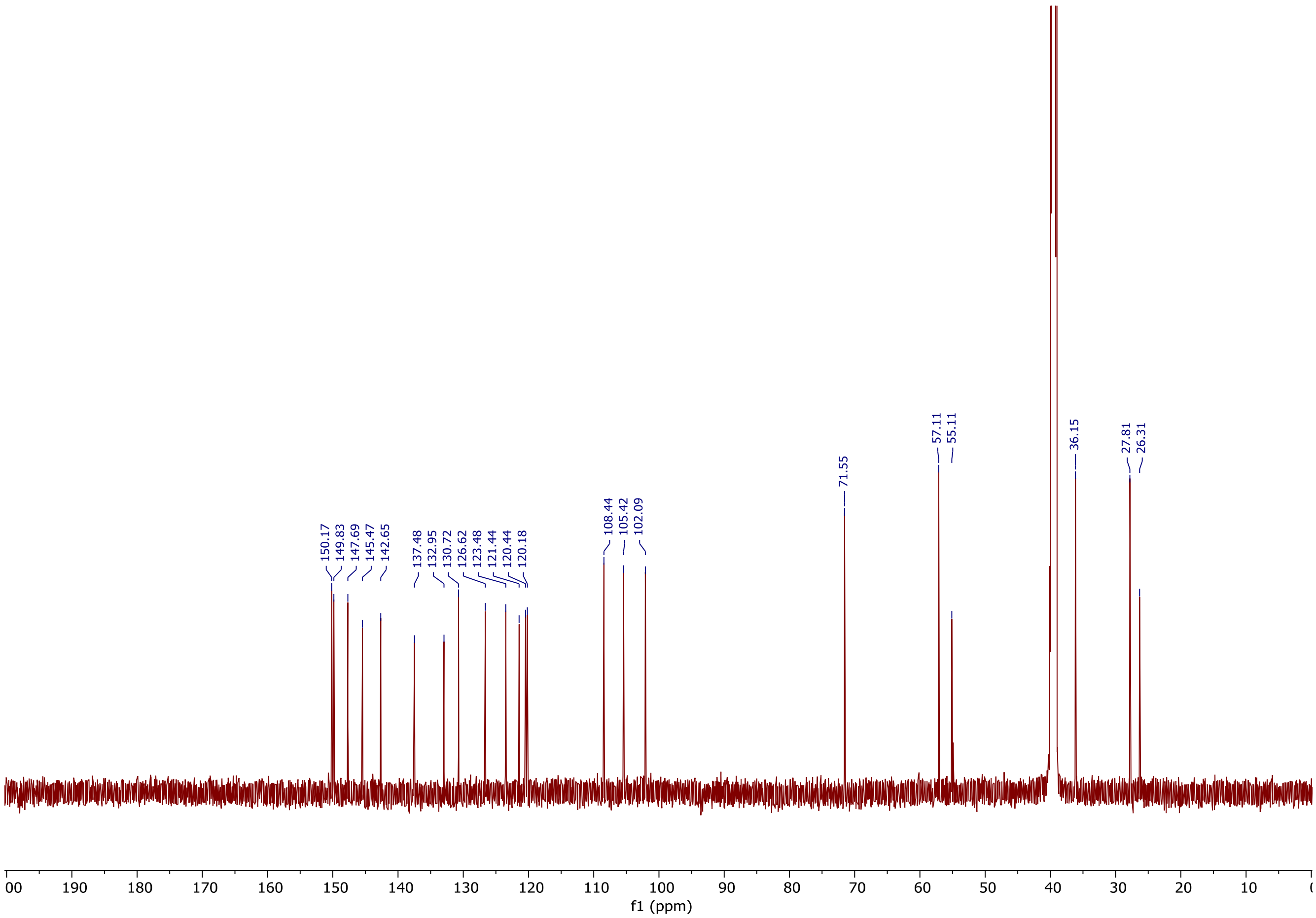
150.17
149.83
147.69
145.47
142.65
137.48
132.95
130.72
126.62
123.48
121.44
120.44
120.18
108.44
105.42
102.09
71.55
57.11
55.11
36.15
27.81
26.31
f1 (ppm)

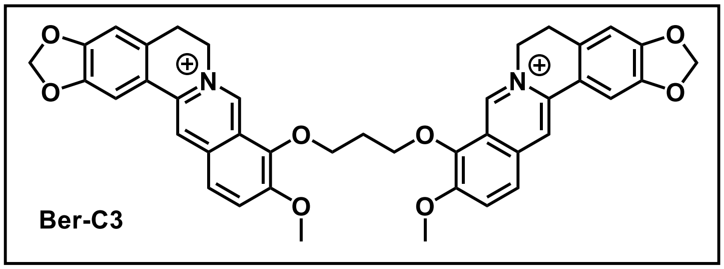

Ber-C3

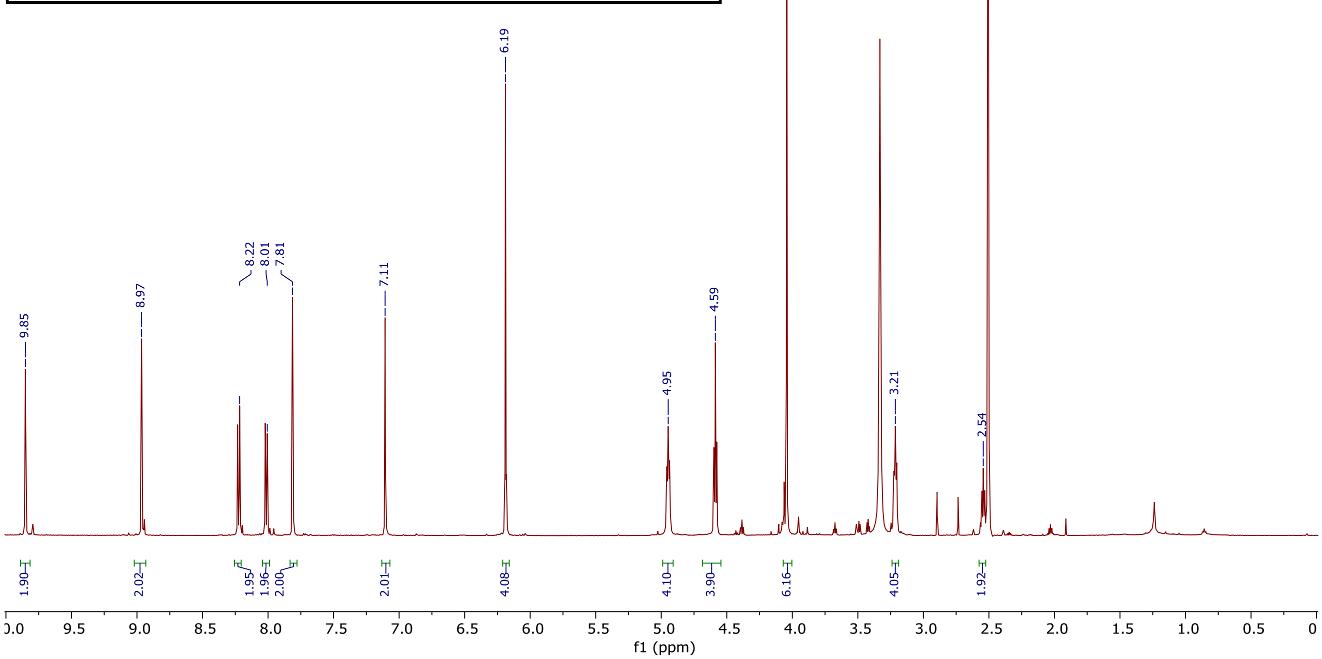

9.85
8.97
8.22
8.01
7.81
7.11
6.19
4.95
4.59
4.04
3.21
2.54
1.90
2.02
1.95
1.96
2.00
2.01
4.08
4.10
3.90
6.16
4.05
1.92
9.5 9.0 8.5 8.0 7.5 7.0 6.5 6.0 5.5 5.0 4.5 4.0 3.5 3.0 2.5 2.0 1.5 1.0 0.5
f1 (ppm)

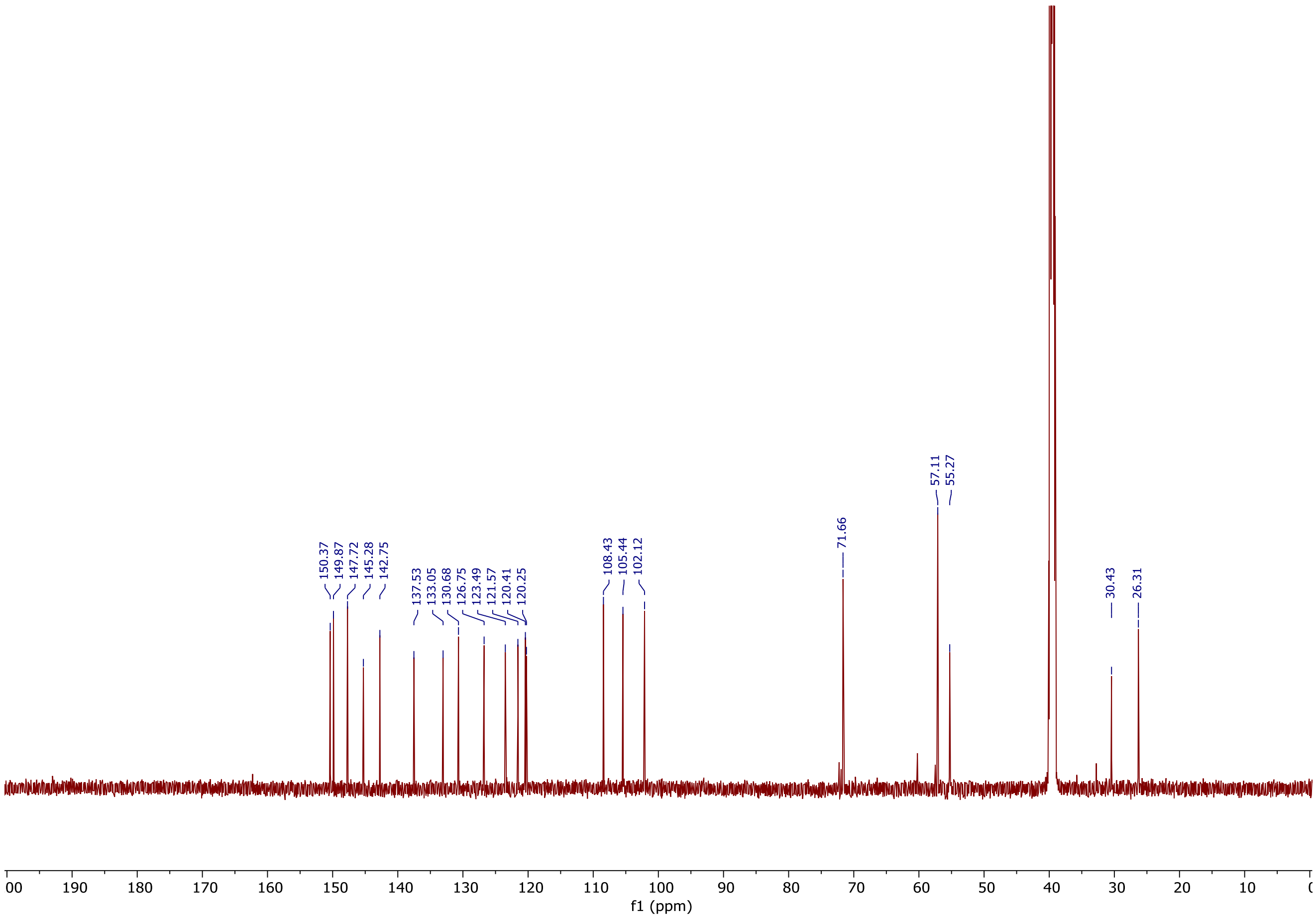

150.37
149.87
147.72
145.28
142.75
137.53
133.05
130.68
126.75
123.49
121.57
120.41
120.25
108.43
105.44
102.12
71.66
57.11
55.27
30.43
26.31
190 180 170 160 150 140 130 120 110 100 90 80 70 60 50 40 30 20 10
f1 (ppm)

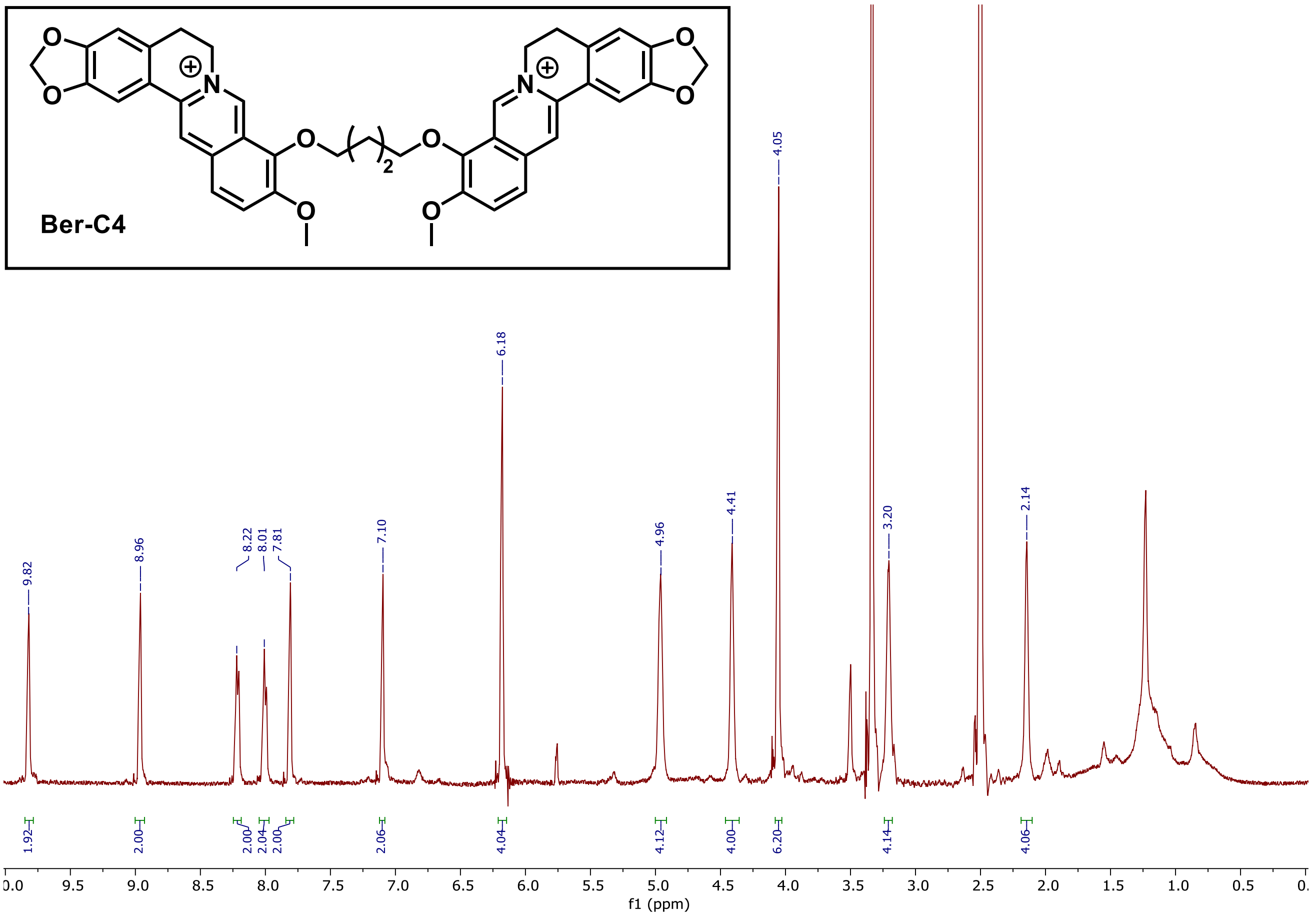

Ber-C4
9.82
8.96
8.22
8.01
7.81
7.10
6.18
4.96
4.41
4.05
3.20
2.14
1.92
2.00
2.00
2.04
2.00
2.06
4.04
4.12
4.00
6.20
4.14
4.06
f1 (ppm)

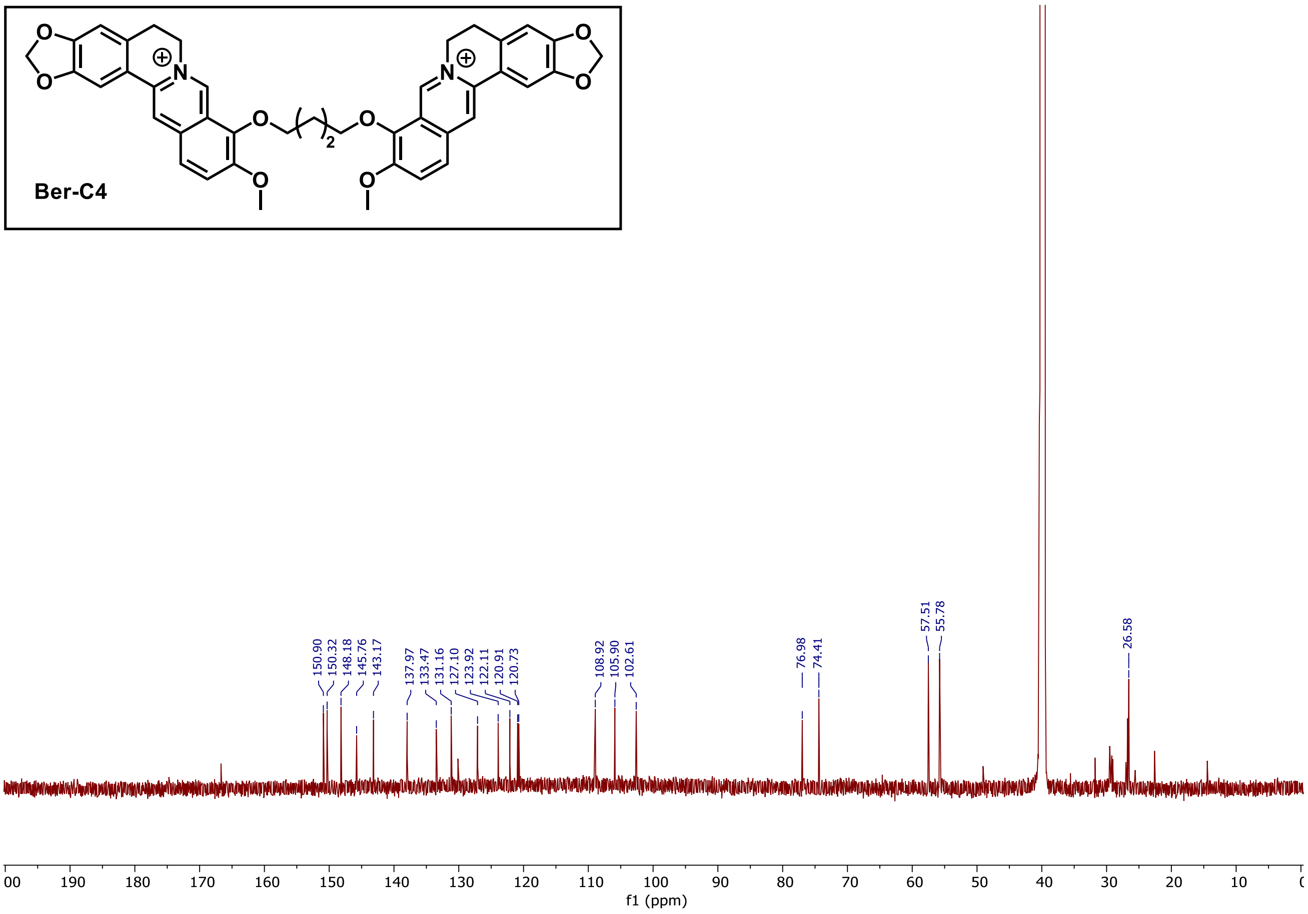

Ber-C4
150.90
150.32
148.18
145.76
143.17
137.97
133.47
131.16
127.10
123.92
122.11
120.91
120.73
108.92
105.90
102.61
76.98
74.41
57.51
55.78
26.58
f1 (ppm)

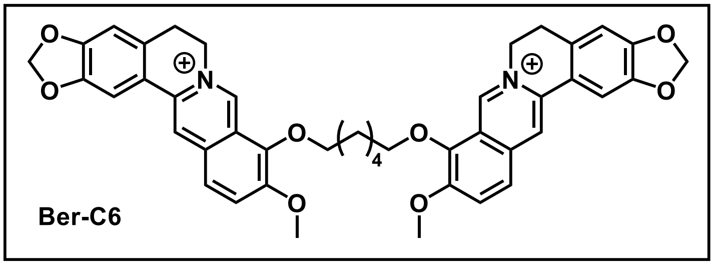

Ber-C6

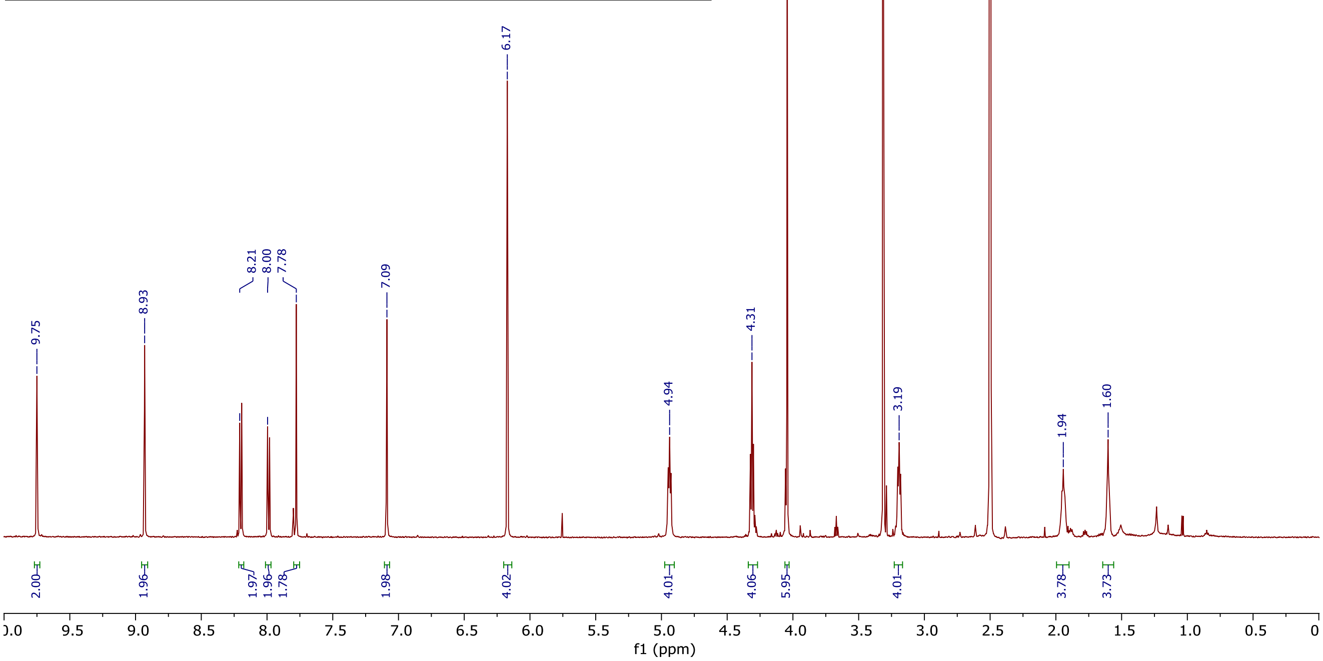

9.75
8.93
8.21
8.00
7.78
7.09
6.17
4.94
4.31
4.04
3.19
1.94
1.60
2.00
1.96
1.97
1.96
1.78
1.98
4.02
4.01
4.06
5.95
4.01
3.78
3.73
f1 (ppm)

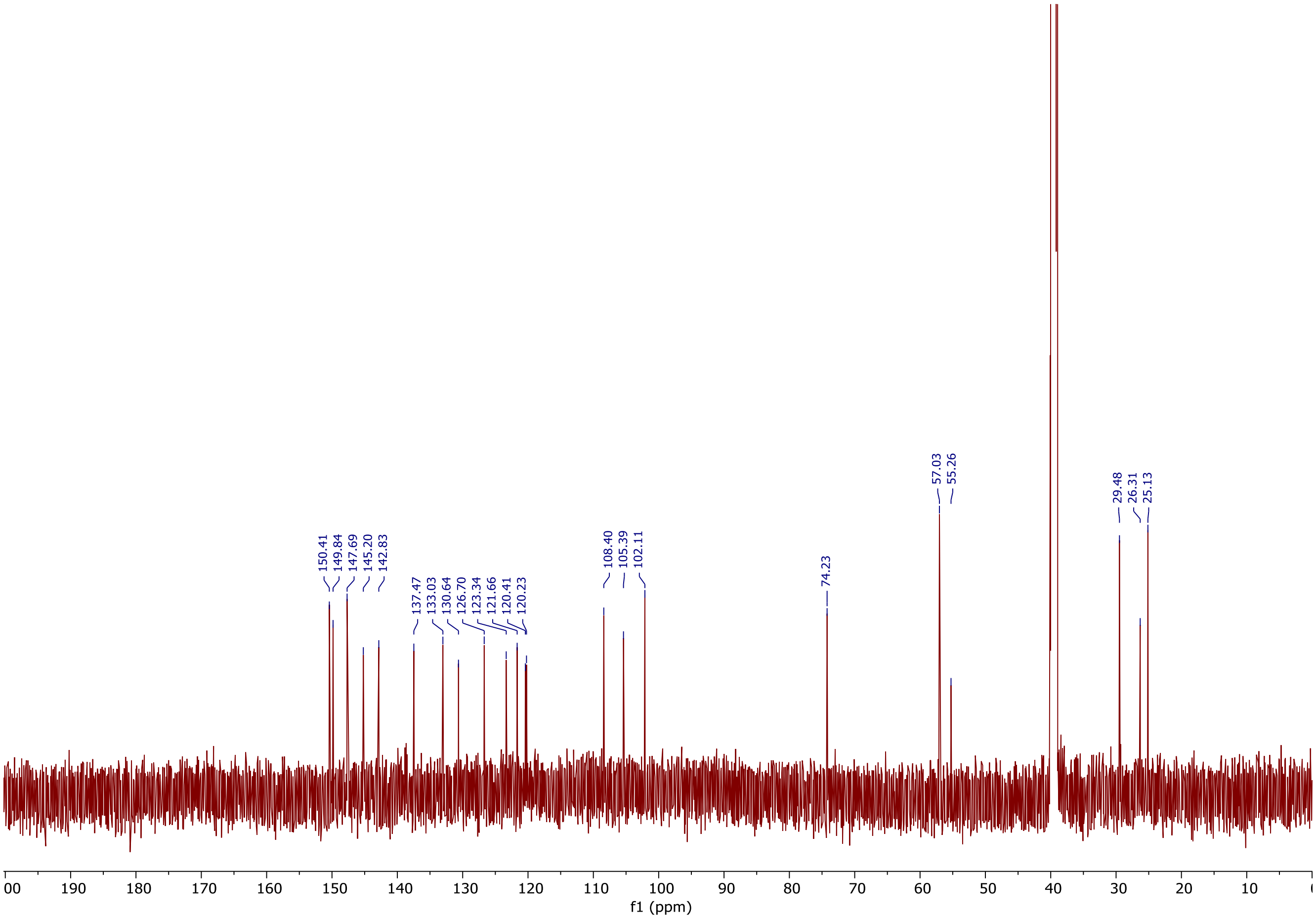

150.41
149.84
147.69
145.20
142.83
137.47
133.03
130.64
126.70
123.34
121.66
120.41
120.23
108.40
105.39
102.11
74.23
57.03
55.26
29.48
26.31
25.13
f1 (ppm)

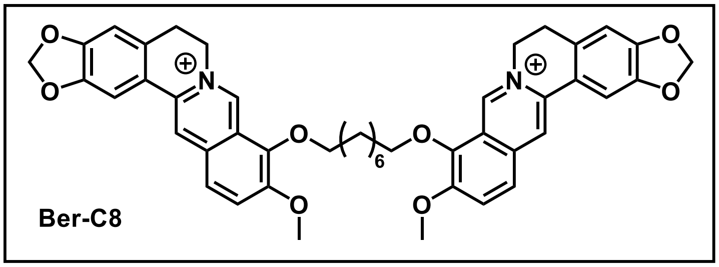
Ber-C8

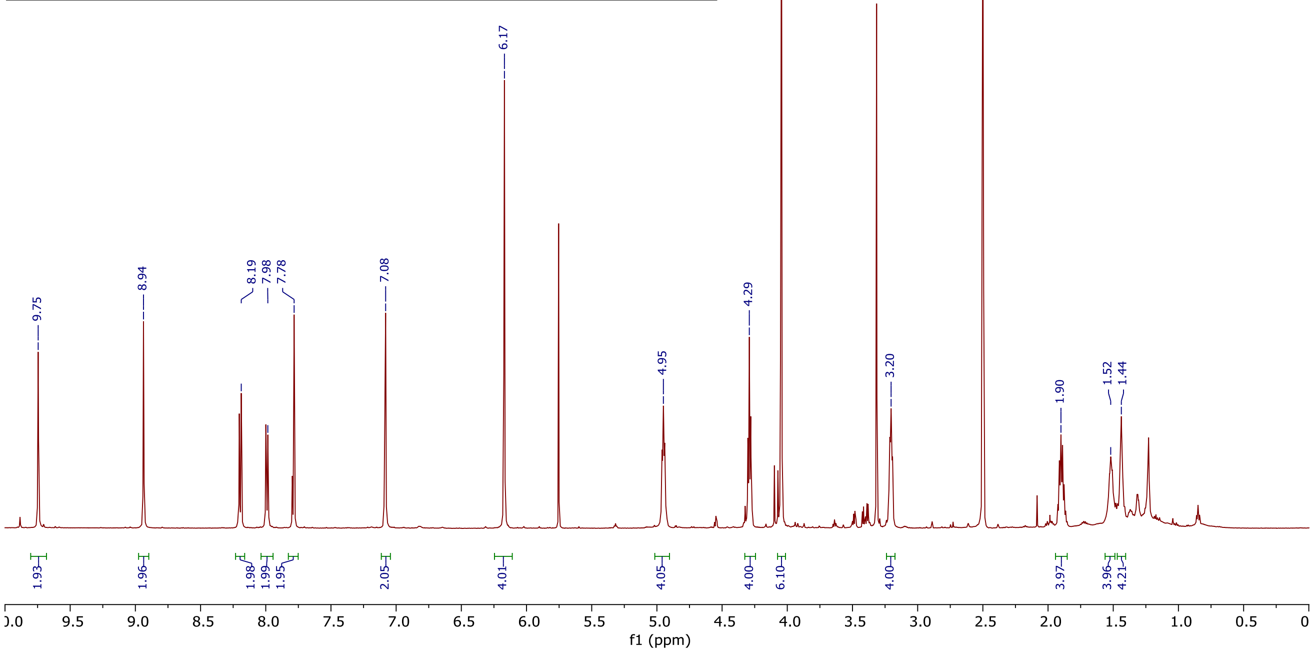
9.75
8.94
8.19
7.98
7.78
7.08
6.17
4.95
4.29
4.04
3.20
2.50 DMSO
1.90
1.52
1.44
1.93
1.96
1.98
1.99
1.95
2.05
4.01
4.05
4.00
6.10
4.00
3.97
3.96
4.21
f1 (ppm)

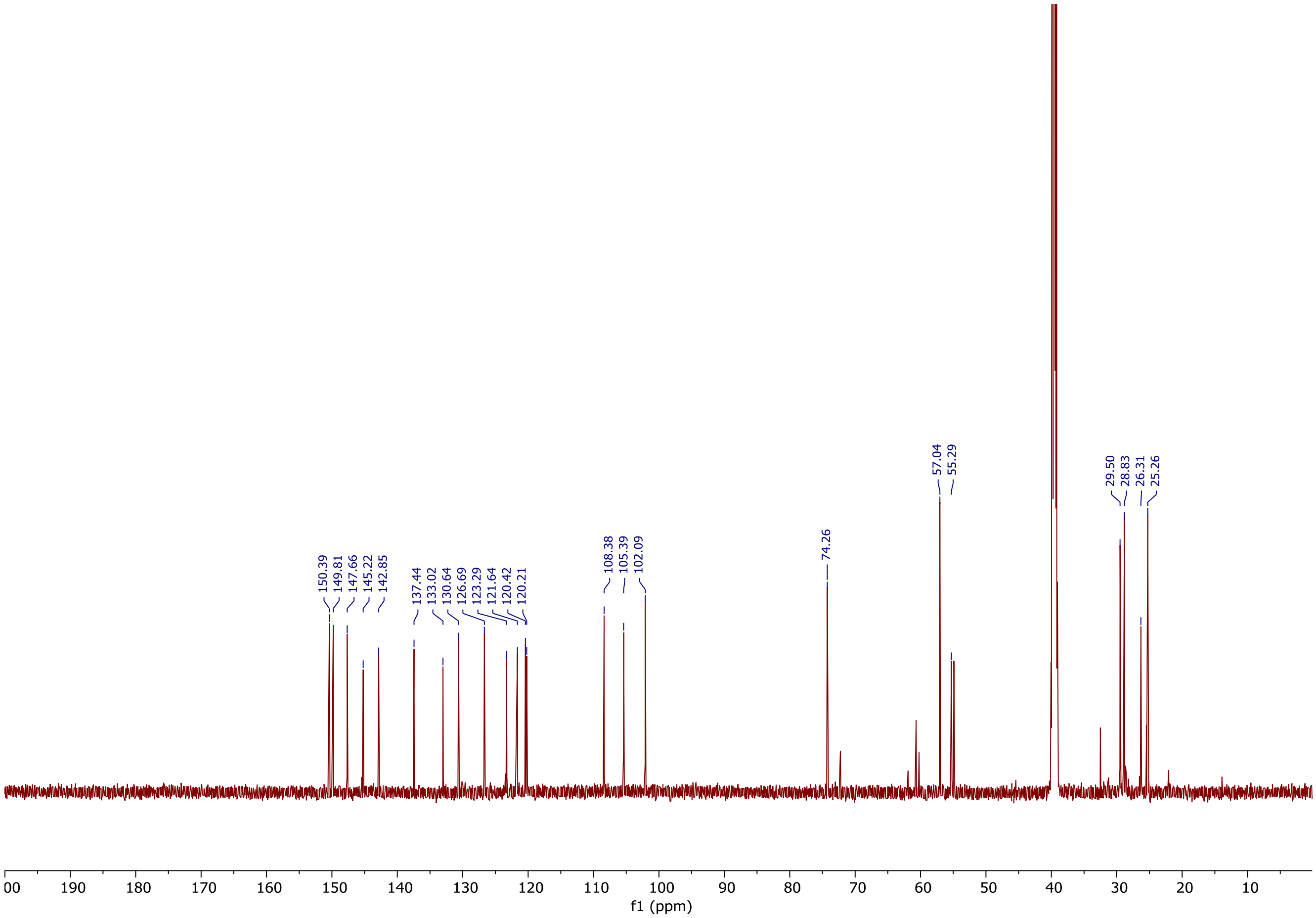
150.39
149.81
147.66
145.22
142.85
137.44
133.02
130.64
126.69
123.29
121.64
120.42
120.21
108.38
105.39
102.09
74.26
57.04
55.29
29.50
28.83
26.31
25.26
f1 (ppm)

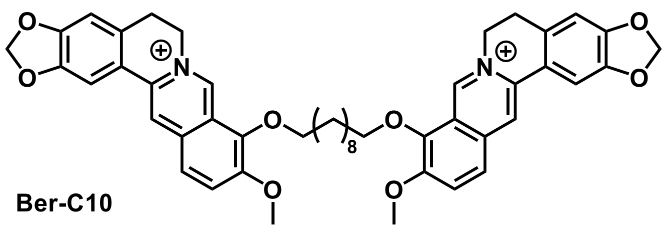
N
O
8

Ber-C10

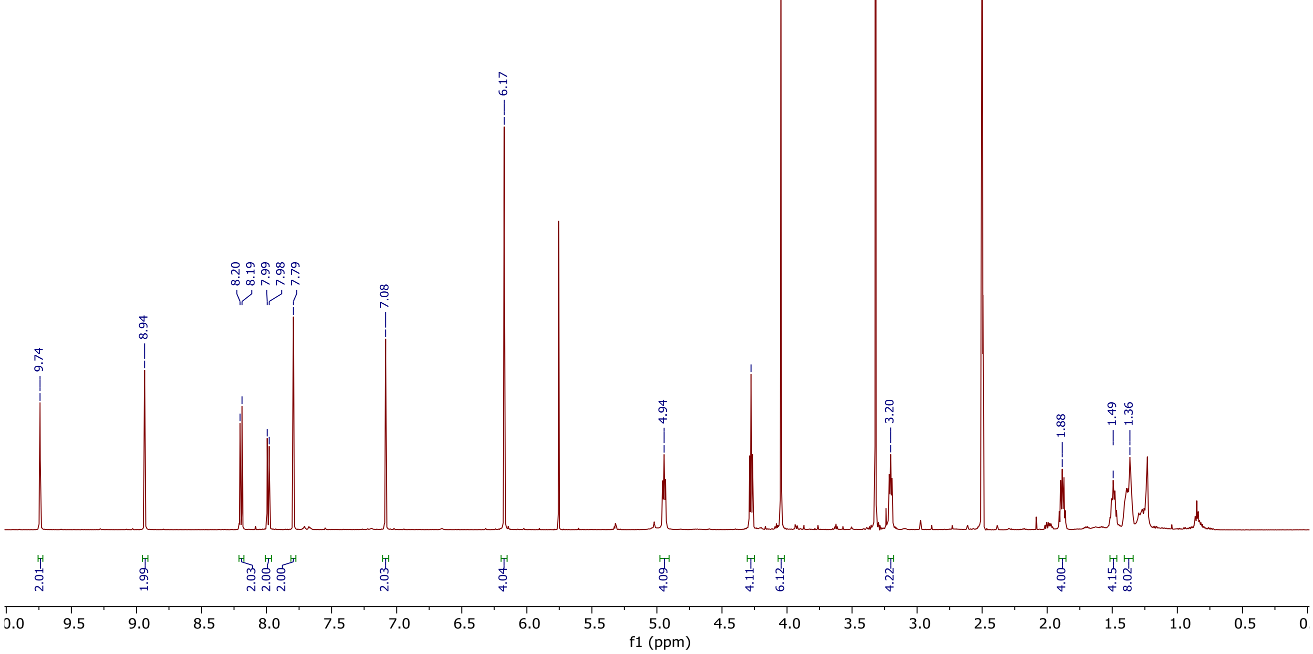
9.74
8.94
8.20
8.19
7.99
7.98
7.79
7.08
6.17
4.94
4.28
4.05
3.20
1.88
1.49
1.36
2.01
1.99
2.03
2.00
2.00
2.03
4.04
4.09
4.11
6.12
4.22
4.00
4.15
8.02
f1 (ppm)

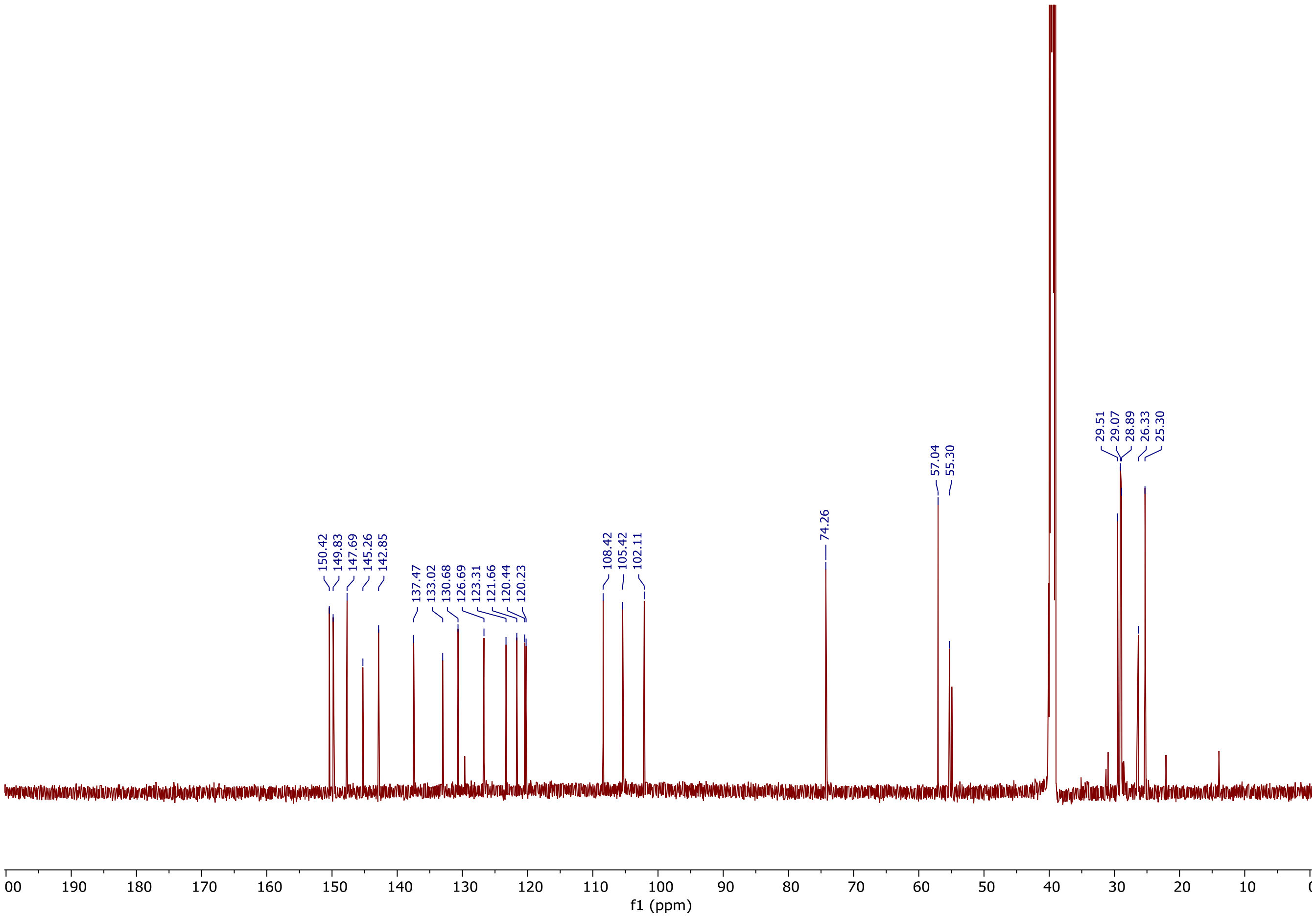
150.42
149.83
147.69
145.26
142.85
137.47
133.02
130.68
126.69
123.31
121.66
120.44
120.23
108.42
105.42
102.11
74.26
57.04
55.30
29.51
29.07
28.89
26.33
25.30
f1 (ppm)

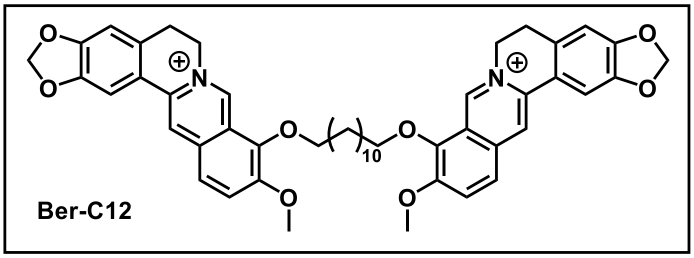
Ber-C12
10

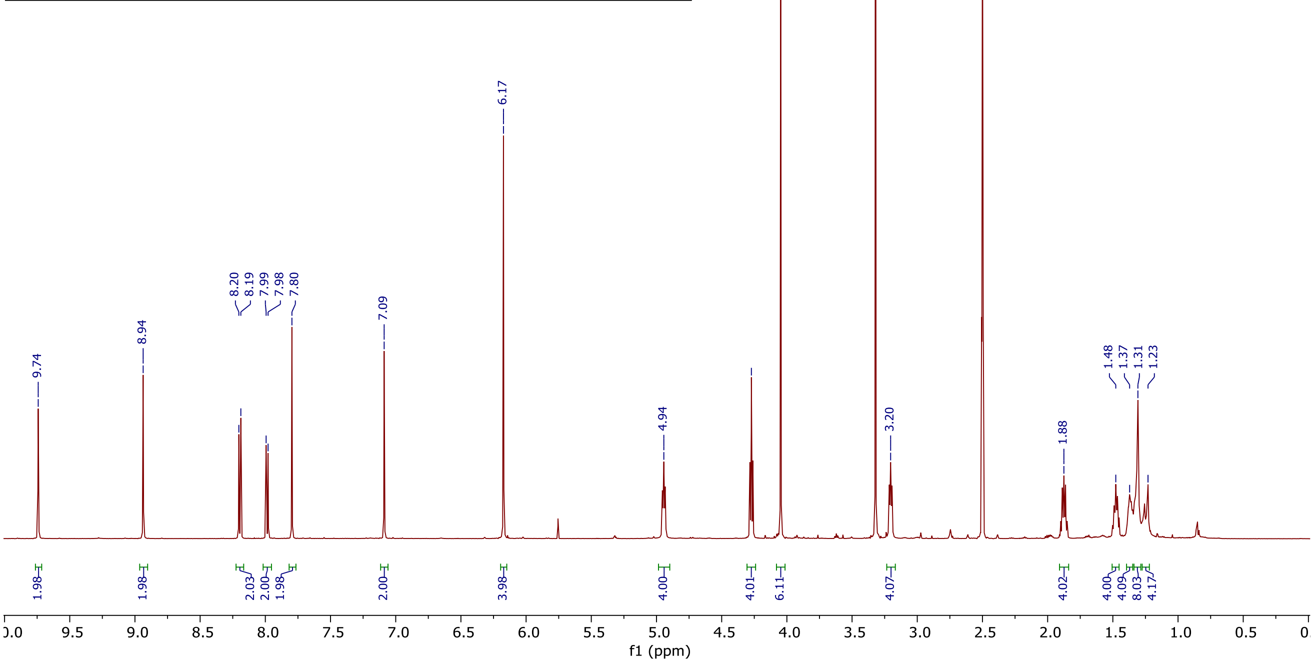
9.74
8.94
8.20
8.19
7.99
7.98
7.80
7.09
6.17
4.94
4.27
4.05
3.20
2.50 DMSO
1.88
1.48
1.37
1.31
1.23
1.98
1.98
2.03
2.00
1.98
2.00
3.98
4.00
4.01
6.11
4.07
4.02
4.00
4.09
8.03
4.17
f1 (ppm)

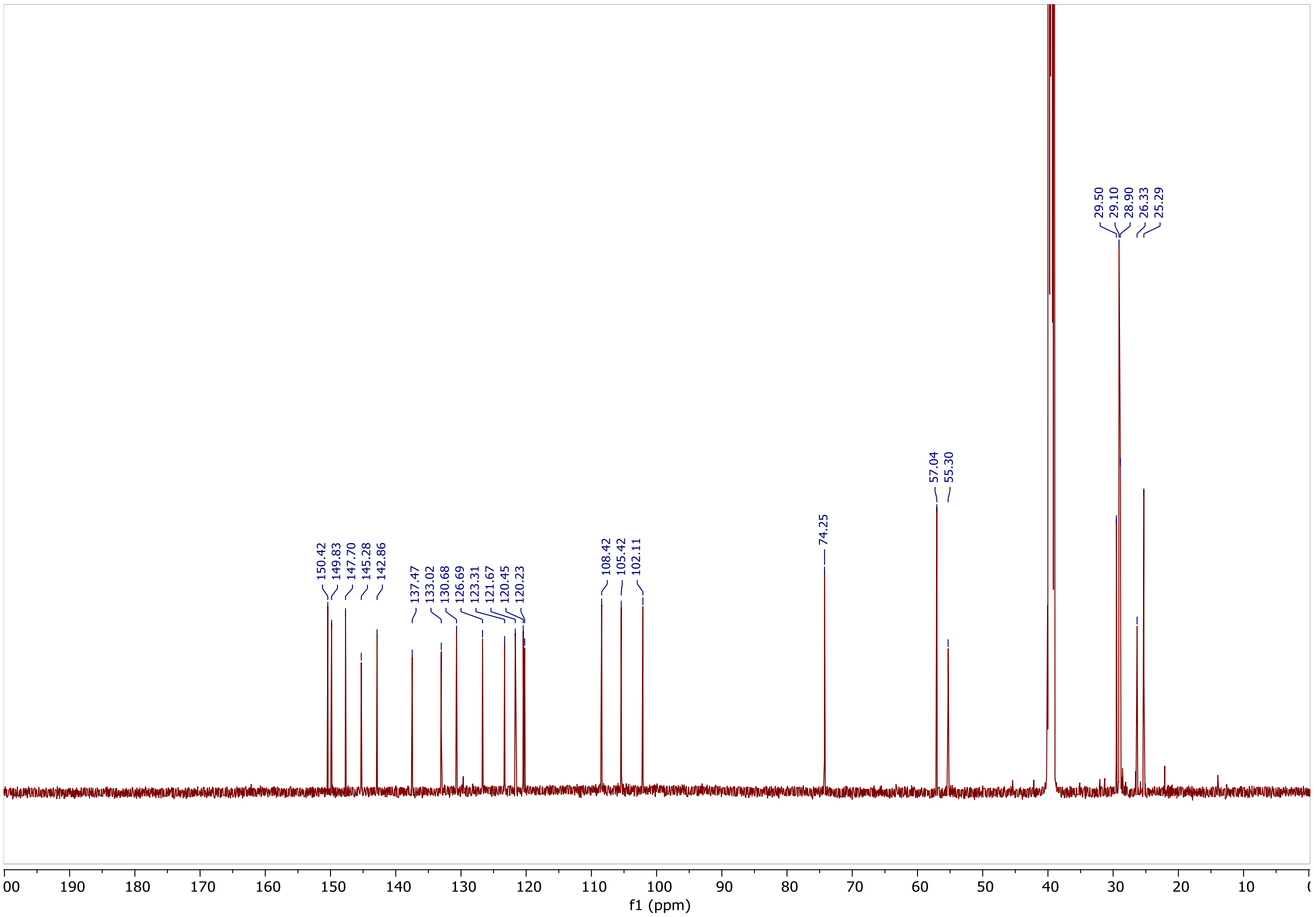
150.42
149.83
147.70
145.28
142.86
137.47
133.02
130.68
126.69
123.31
121.67
120.45
120.23
108.42
105.42
102.11
74.25
57.04
55.30
29.50
29.10
28.90
26.33
25.29
f1 (ppm)

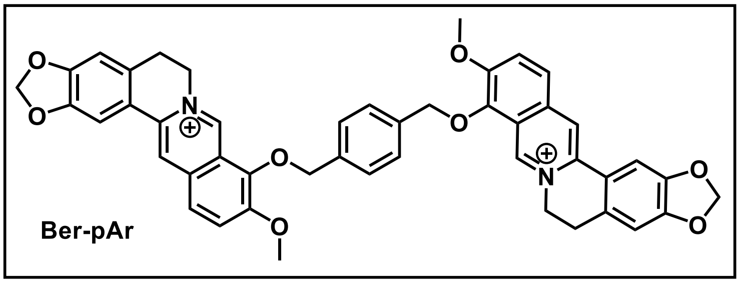
Ber-pAr

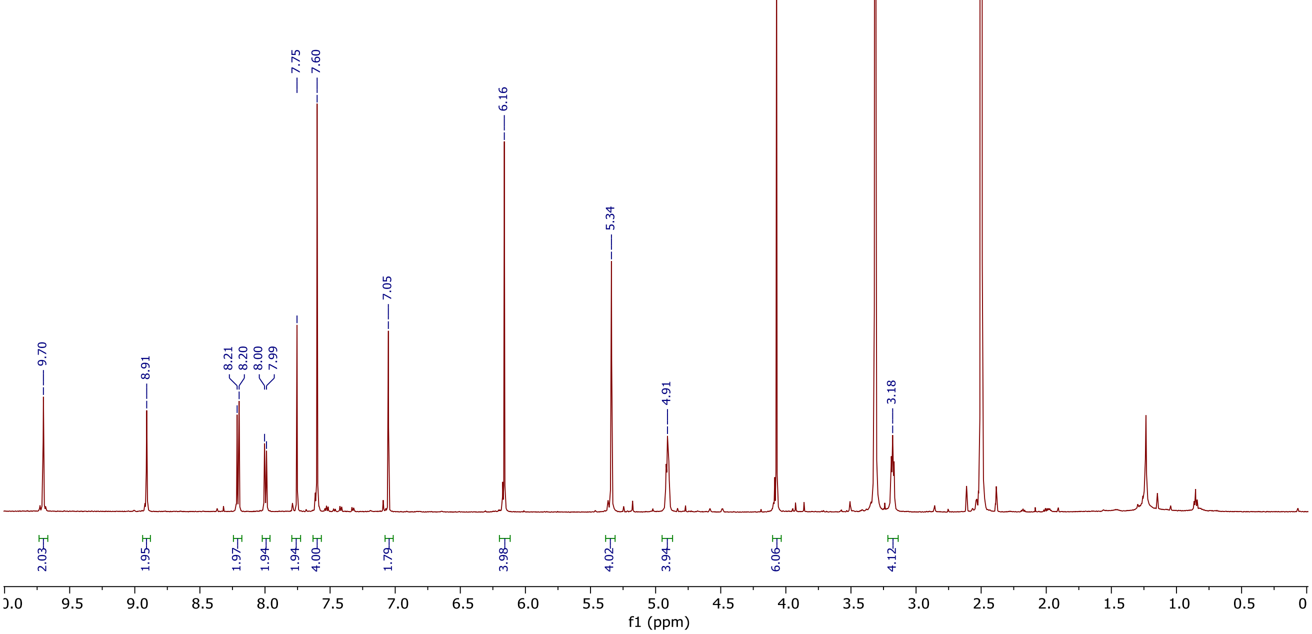
9.70
8.91
8.21
8.20
8.00
7.99
7.75
7.60
7.05
6.16
5.34
4.91
4.07
3.18
2.03
1.95
1.97
1.94
1.94
4.00
1.79
3.98
4.02
3.94
6.06
4.12
f1 (ppm)

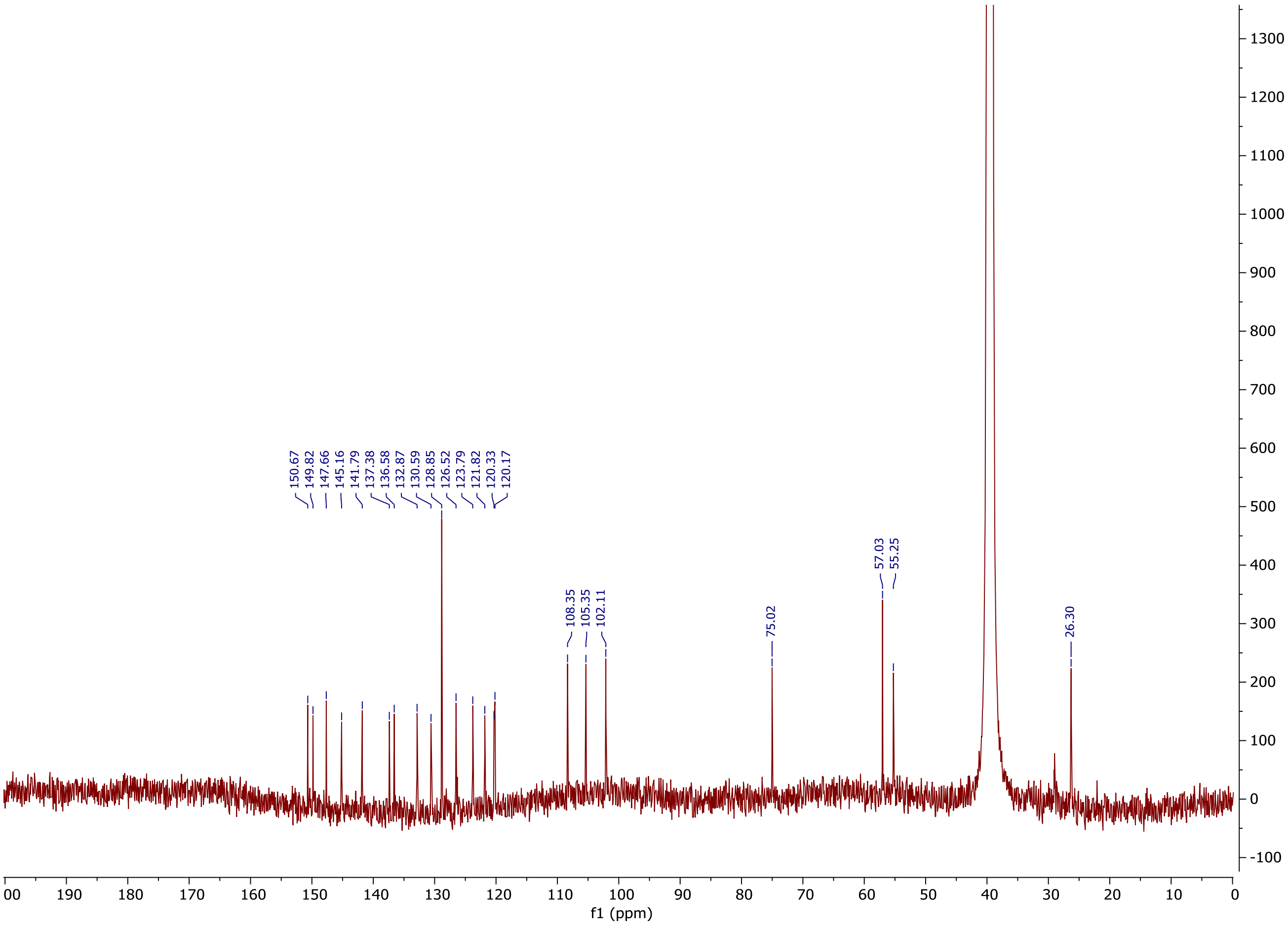
150.67
149.82
147.66
145.16
141.79
137.38
136.58
132.87
130.59
128.85
126.52
123.79
121.82
120.33
120.17
108.35
105.35
102.11
75.02
57.03
55.25
26.30
f1 (ppm)

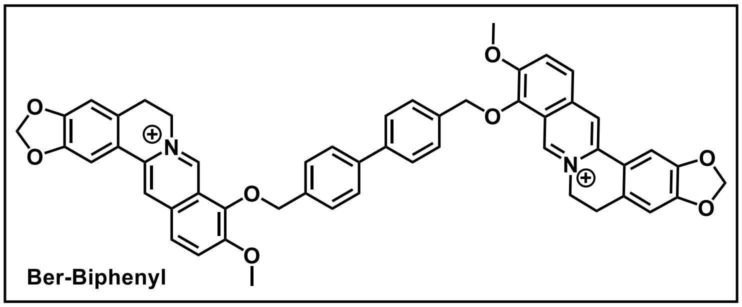

Ber-Biphenyl

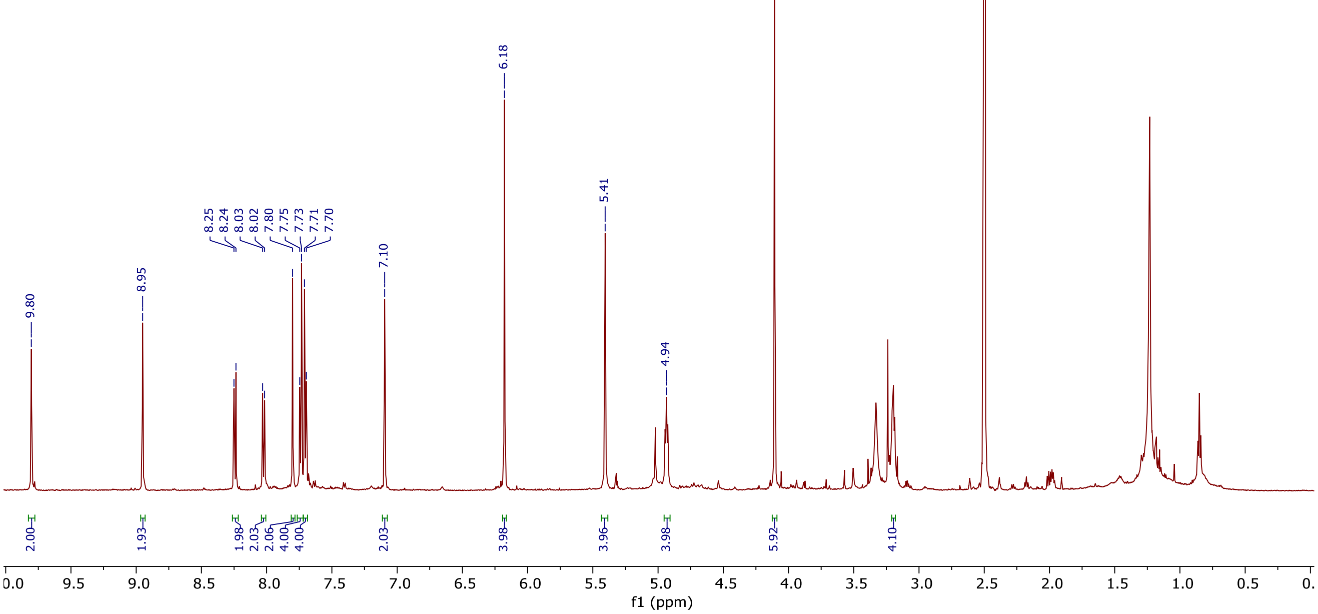

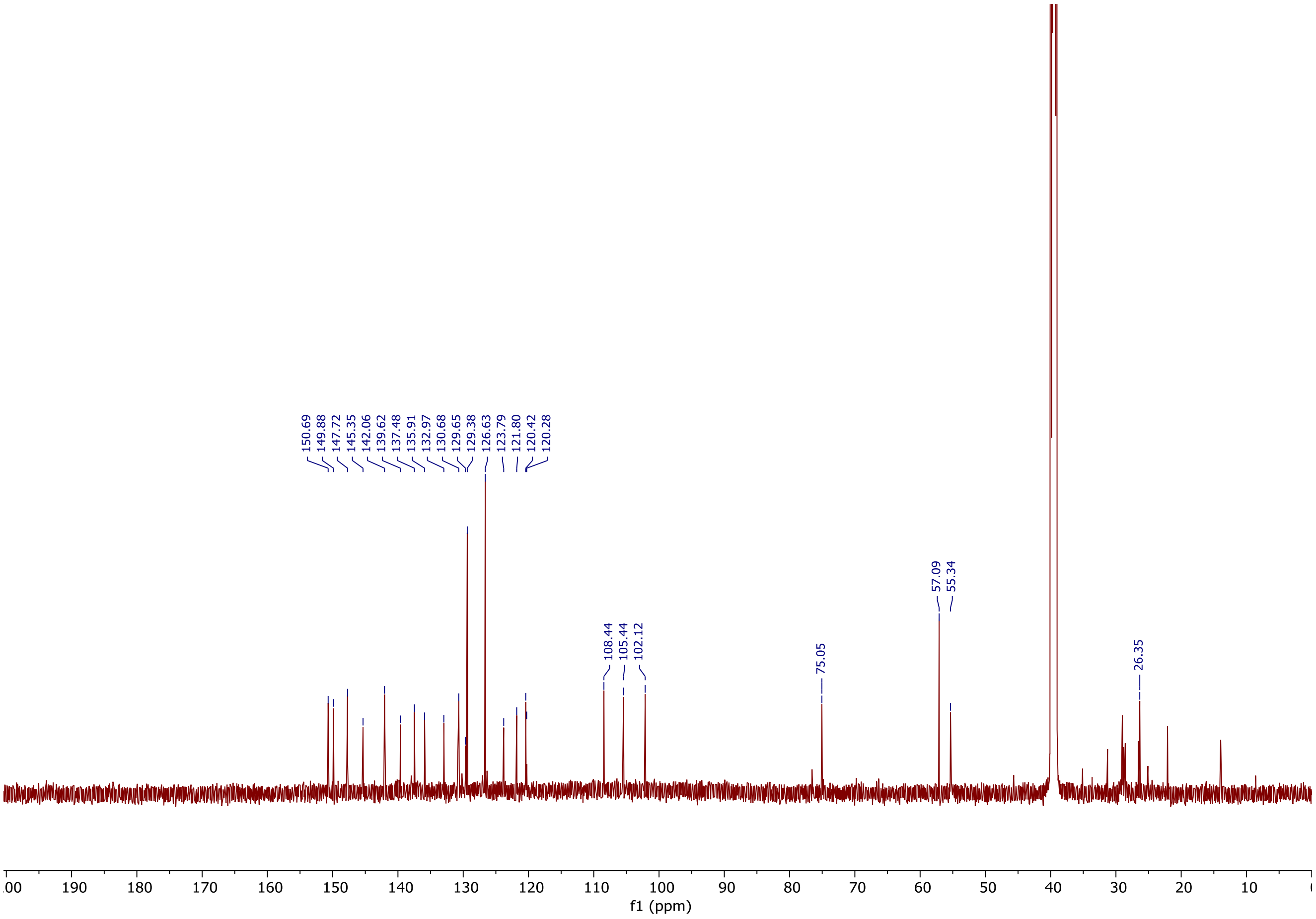

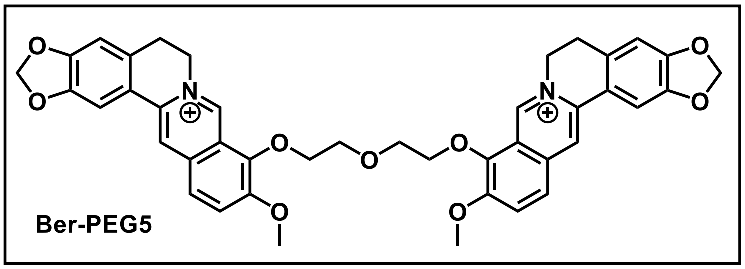
Ber-PEG5

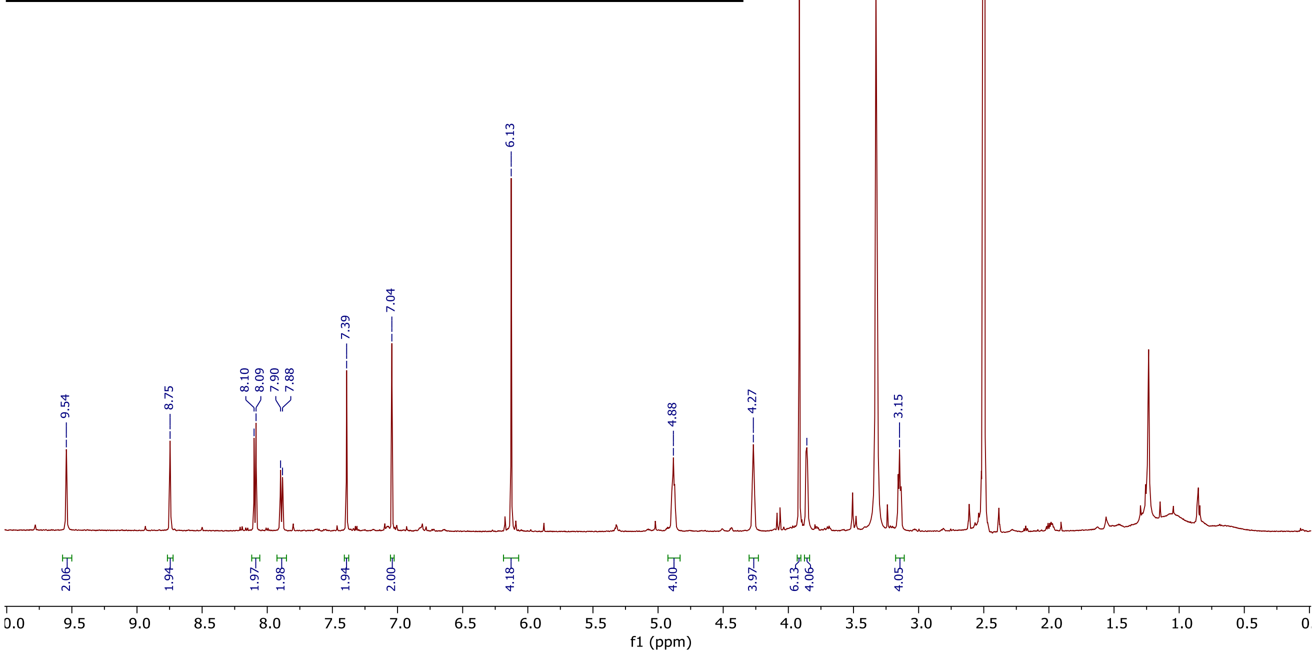
9.54
8.75
8.10
8.09
7.90
7.88
7.39
7.04
6.13
4.88
4.27
3.92
3.86
3.15
2.06
1.94
1.97
1.98
1.94
2.00
4.18
4.00
3.97
6.13
4.06
4.05
0.0 9.5 9.0 8.5 8.0 7.5 7.0 6.5 6.0 5.5 5.0 4.5 4.0 3.5 3.0 2.5 2.0 1.5 1.0 0.5 0
f1 (ppm)

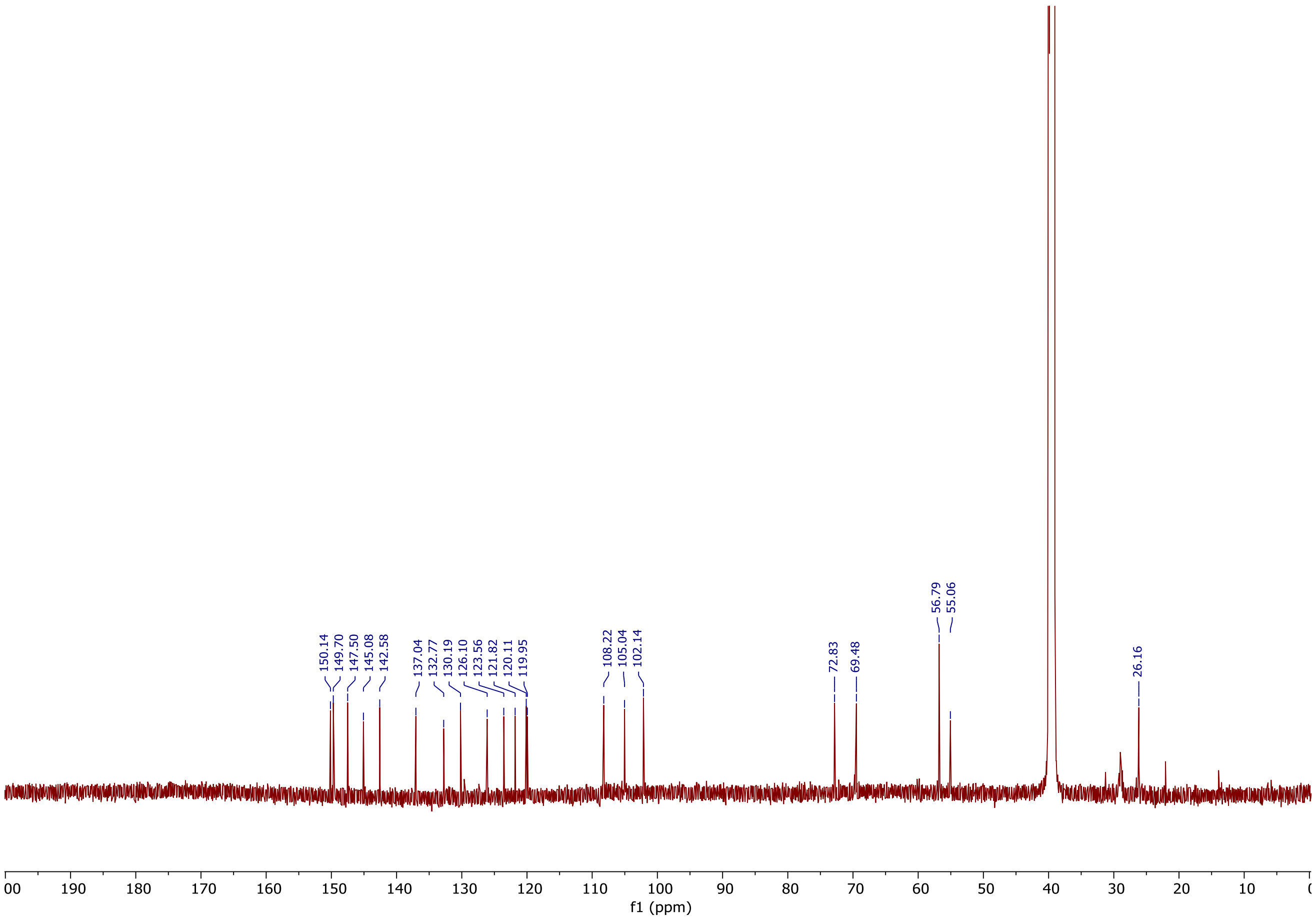
150.14
149.70
147.50
145.08
142.58
137.04
132.77
130.19
126.10
123.56
121.82
120.11
119.95
108.22
105.04
102.14
72.83
69.48
56.79
55.06
26.16
00 190 180 170 160 150 140 130 120 110 100 90 80 70 60 50 40 30 20 10
f1 (ppm)

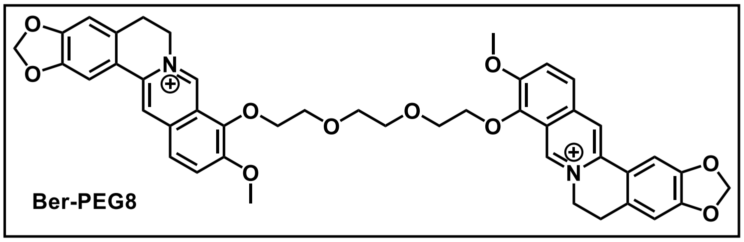
Ber-PEG8

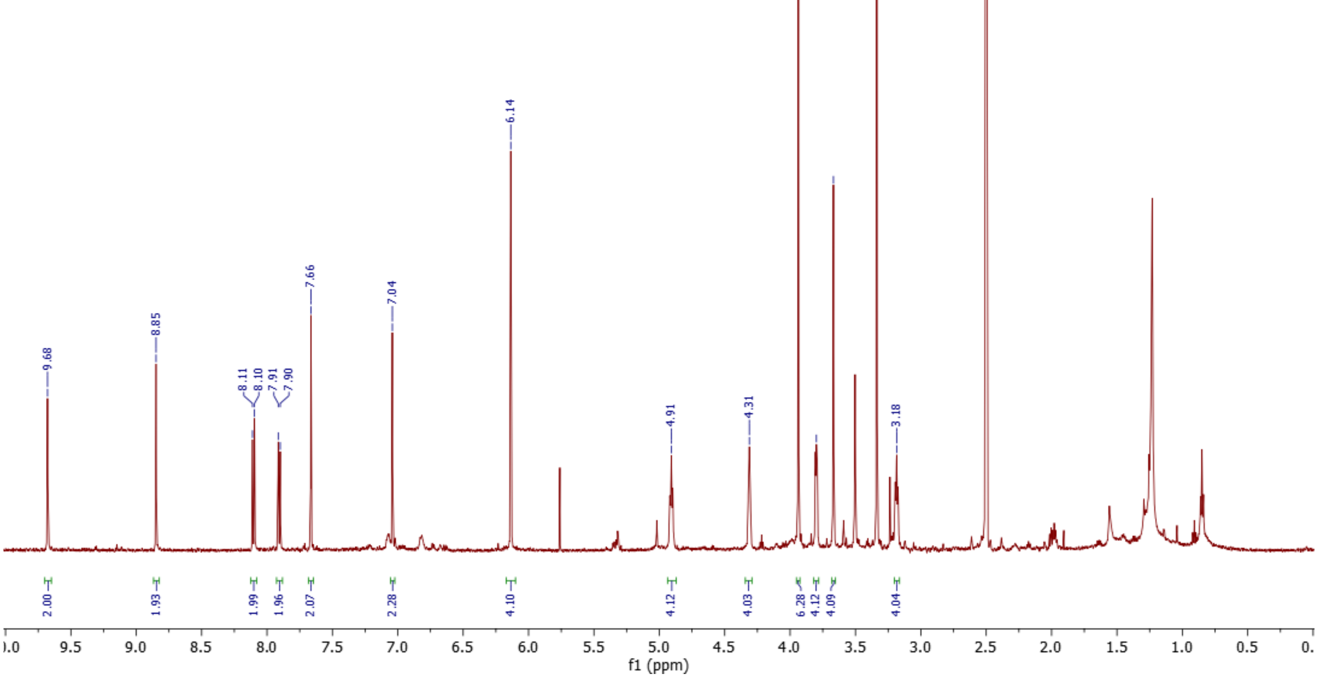
3.94
3.80
3.67
6.14
9.68
8.85
8.11
8.10
7.91
7.90
7.66
7.04
4.91
4.31
3.18
2.00
1.93
1.99
1.96
2.07
2.28
4.10
4.12
4.03
6.28
4.12
4.09
4.04
9.5 9.0 8.5 8.0 7.5 7.0 6.5 6.0 5.5 5.0 4.5 4.0 3.5 3.0 2.5 2.0 1.5 1.0 0.5
f1 (ppm)

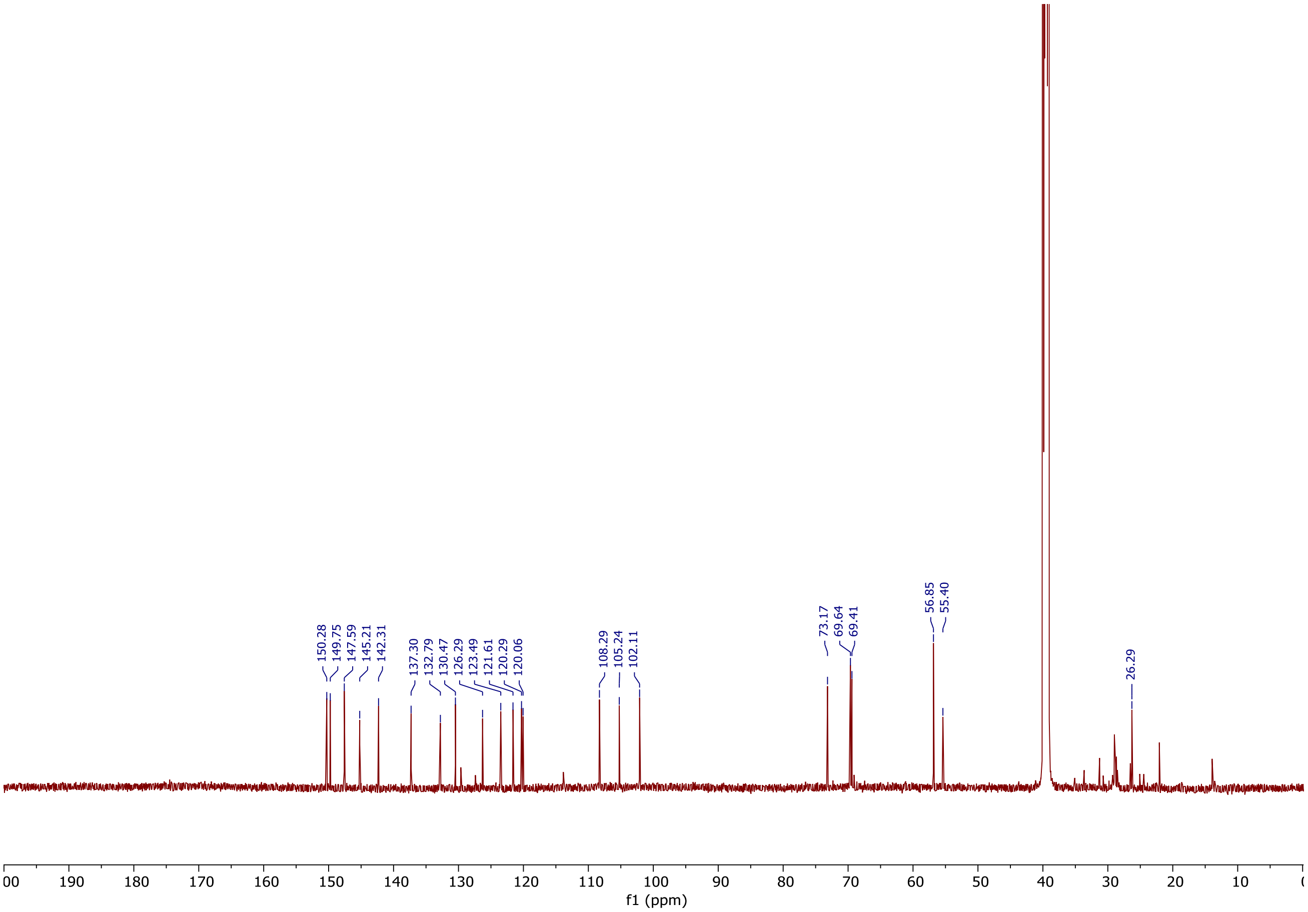
150.28
149.75
147.59
145.21
142.31
137.30
132.79
130.47
126.29
123.49
121.61
120.29
120.06
108.29
105.24
102.11
73.17
69.64
69.41
56.85
55.40
26.29
190 180 170 160 150 140 130 120 110 100 90 80 70 60 50 40 30 20 10
f1 (ppm)
